# Response Trajectories and Temporal Trends of Viloxazine Treatment for Young People With ADHD: A Meta-Analysis

**DOI:** 10.1001/jamanetworkopen.2024.45885

**Published:** 2024-11-19

**Authors:** Chia-Ling Yu, Yu-Chen Kao, Trevor Thompson, Brendon Stubbs, Ping-Tao Tseng, Chih-Wei Hsu, Fu-Chi Yang, Yu-Kang Tu, Tien-Wei Hsu, Chih-Sung Liang

**Affiliations:** 1 Department of Pharmacy, Chang Gung Memorial Hospital, Linkou, Taoyuan, Taiwan; 2 Department of Psychiatry, Tri-Service General Hospital, National Defense Medical Centre, Taipei, Taiwan; 3 Department of Psychiatry, Beitou Branch, Tri-Service General Hospital, Taipei, Taiwan; 4 Centre for Chronic Illness and Ageing, University of Greenwich, London, United Kingdom; 5 Institute of Psychiatry, Psychology and Neuroscience, King’s College London, London, United Kingdom; 6 Institute of Biomedical Sciences, National Sun Yat-sen University, Kaohsiung, Taiwan; 7 Department of Psychology, College of Medical and Health Science, Asia University, Taichung, Taiwan; 8 Prospect Clinic for Otorhinolaryngology & Neurology, Kaohsiung, Taiwan; 9 Institute of Precision Medicine, National Sun Yat-sen University, Kaohsiung City, Taiwan; 10 Department of Psychiatry, Kaohsiung Chang Gung Memorial Hospital, Kaohsiung, Taiwan; 11 Department of Neurology, Tri-Service General Hospital, National Defense Medical Centre, Taipei, Taiwan; 12 Institute of Health Data Analytics & Statistics, College of Public Health, National Taiwan University, Taipei, Taiwan; 13 Department of Psychiatry, E-Da Dachang Hospital, I-Shou University, Kaohsiung, Taiwan; 14 Department of Psychiatry, E-Da Hospital, I-Shou University, Kaohsiung, Taiwan

## Abstract

**Importance:**

Viloxazine is a novel nonstimulant medication approved for the treatment of attention-deficit/hyperactivity disorder (ADHD).

**Objectives:**

To investigate the whether viloxazine is associated with effective and acceptable outcomes when treating children and adolescents with ADHD and to evaluate these outcomes’ associations with viloxazine doses and duration of treatment.

**Data Sources:**

The MEDLINE, Cochrane Central Register of Controlled Trials (CENTRAL), Embase, PsycINFO, and ClinicalTrial.gov databases were searched from database inception to June 23, 2024.

**Study Selection:**

Two reviewers independently screened for double-blind, fixed-dose randomized clinical trials (RCTs) that compared viloxazine with placebo for pediatric patients with ADHD.

**Data Extraction and Synthesis:**

The study followed the Preferred Reporting Items for Systematic Reviews and Meta-Analyses reporting guideline. Data extraction was completed independently by 2 authors and cross-checked for errors. Random-effects pairwise and dose-response meta-analyses were conducted.

**Main Outcomes and Measures:**

The primary outcome was the improvement of ADHD symptoms (measured by ADHD Rating Scale–5), and the secondary outcomes were all-cause discontinuation, dropout due to adverse effects, and serious adverse effects.

**Results:**

A total of 5 dose-response RCTs were included, with 1560 participants (1011 [64.8%] male; mean [SD] age, 10.6 [6.7] years). Viloxazine was associated with better outcomes in ADHD treatment compared with placebo (mean difference, 5.47 points; 95% CI, 4.03-6.91 points). The dose-response curve was bell-shaped, suggesting that doses greater than 400 mg or greater than 7 mg/kg might not be associated with more efficacy. The temporal trends analysis showed ascent curves tapering off at approximately weeks 4 to 6. The curve for 100 mg/d declined more rapidly, while the curves for 200 mg/d and 400 mg/d declined more gradually. The overall discontinuation rate due to adverse effects was 4.15% in the viloxazine group (45 of 1084), while viloxazine compared with placebo was associated with 2.48-fold higher risk of discontinuation due to adverse effects (risk ratio, 2.48; 95% CI, 1.26-4.88).

**Conclusions and relevance:**

In this meta-analysis, viloxazine was associated with better efficacy in treating children and adolescents with ADHD than placebo. A moderate dose (200-400 mg or 6-8 mg/kg) may provide optimal treatment outcomes. Future studies are warranted to assess the long-term effect of viloxazine. Viloxazine was relatively well tolerated for children and adolescents with ADHD.

## Key Points

**Question:**

Is viloxazine associated with effective and acceptable outcomes among children and adolescents with attention-deficit/hyperactivity disorder (ADHD), and how are these outcomes associated with dose and treatment duration?

**Findings:**

In this meta-analysis that included 5 fixed-dose randomized clinical trials and 1560 participants, viloxazine was associated with better efficacy in treating ADHD symptoms compared with placebo, showing bell-shaped response trajectories.

**Meaning:**

In this study, viloxazine was well-tolerated and associated with improvements in ADHD symptoms, and a moderate dose (200-400 mg or 6-8 mg/kg) appeared to provide the best treatment outcomes.

## Teaser

This meta-analysis evaluates whether viloxazine, a novel nonstimulant medication, is associated with symptom improvements among children and adolescents with attention-deficit/hyperactivity disorder (ADHD).

## Introduction

Attention-deficit/hyperactivity disorder (ADHD) is a neurodevelopmental disorder characterized by persistent patterns of inattention, hyperactivity, and impulsivity that interfere with functioning or development. It is commonly diagnosed in childhood but can continue to affect individuals’ daily lives into adulthood.^1,2^ The prevalence of ADHD varies globally, with studies indicating significant differences across regions. In children, the global prevalence is estimated to be approximately 5% to 7%, although it can range from 2% to 17% depending on the diagnostic criteria used and geographical location.^3,4^ Among adults, the prevalence is approximately 2.5%, reflecting the continuation of the disorder beyond childhood in a substantial number of cases.^5^

The mainstay of ADHD treatment is pharmacological intervention and involves the use of stimulant and nonstimulant medications, which have been shown to be effective in managing symptoms of inattention, hyperactivity, and impulsivity.^6^ However, common stimulants, including methylphenidate and amphetamines, increase dopamine and norepinephrine levels in the synaptic cleft, with possible adverse effects (AEs), including elevated blood pressure, decreased appetite, insomnia, risk of dependence, and even psychosis.^7^ Nonstimulant options include atomoxetine, guanfacine, and clonidine. Atomoxetine selectively inhibits the reuptake of norepinephrine and increases its availability in the synaptic cleft.^8^ Guanfacine and clonidine are α-2 adrenergic agonists that modulate the release of norepinephrine.^9^ However, these nonstimulants also cause AEs, such as somnolence, fatigue, nausea, and abdominal pain, which may be intolerable for some patients.^9^ Importantly, approximately 20% to 30% of patients with ADHD do not respond to methylphenidate treatment.^10^ Furthermore, a recent meta-analysis reported that both atomoxetine and guanfacine were less effective in treating ADHD symptoms compared with methylphenidate.^11^ Therefore, there is a need for new-generation medications for the management of ADHD.

Viloxazine has been used clinically in Europe for many years as an antidepressant. It is now approved as a novel nonstimulant medication for the treatment of ADHD, with norepinephrine transporter inhibition, serotonin receptor modulations, and indirect dopamine enhancement properties.^12^ Recently, several large randomized clinical trials (RCTs)^13,14,15,16^ have reported that viloxazine shows significant efficacy compared with placebo in treating ADHD in children and adolescents, and it also has relatively good tolerability. A recent meta-analysis of nonstimulants in adult ADHD^17^ demonstrated that viloxazine might be associated with better efficacy for ADHD compared with atomoxetine and guanfacine. There are currently 2 meta-analyses^18,19^ to investigate the outcomes and AEs of viloxazine in children and adolescents with ADHD. However, the first study^18^ only assessed outcomes based on whether the patient was a treatment responder (>50% symptoms reduction), rather than using continuous variables and calculating the standard mean difference. The second study^19^ only assessed safety as the relative risks of AEs and SAEs, rather than specific types of AEs. Importantly, the dose-response trajectories of viloxazine and its temporal trends of treatment success and AEs are also unclear, and determining optimal dosage is fundamental for informing guideline development.

To fill this gap, we conducted the first dose-response meta-analysis that we know of to investigate the association of the repurposed antidepressant viloxazine with effective and acceptable outcomes in treating children and adolescents with ADHD as well as its dose-response trajectories and temporal trends. Our research findings may help psychiatrists and pediatrists better understand the optimal dosage and treatment trajectory of viloxazine for ADHD.

## Methods

Prior to data analysis, we registered our protocol on PROSPERO (CRD42024553701). The study adhered to the Preferred Reporting Items for Systematic Reviews and Meta-Analyses (PRISMA) reporting guideline.^20^

### Eligibility Criteria

We included fixed-dose RCTs that compared viloxazine with placebo or alternative doses of the drug in children and adolescents (aged 6-17 years) with a diagnosis of ADHD (*Diagnostic and Statistical Manual for Mental Disorders, Fifth Edition*, or* International Classification of Diseases, 11th Edition*). The placebo group consisted of a capsule or tablet with no pharmacological effect and was indistinguishable in appearance from viloxazine. It did not contain any substances with pharmacological effects (eg, low-dose viloxazine) or include any other nonpharmacological treatments (eg, behavioral therapy). We excluded studies (1) using flexible dose, (2) including adults with ADHD, or (3) focusing on other comorbidities, such as Tourette syndrome.

### Data Sources and Study Selection

We searched MEDLINE, Cochrane Central Register of Controlled Trials, Embase, PsycINFO, ClinicalTrial.gov, and PubMed databases from inception to June 23, 2024, without language restrictions (eAppendix 1 in Supplement 1). Two reviewers (C.-L. Y. and T.-W. H.) and independently screened titles, abstracts, and full-text articles of selected records to confirm eligibility. Title, abstract, and full-text screening required agreement between reviewers. Reviewers resolved disagreements by discussion and, if necessary, by consultation with a third senior author (C.-S. L.).

### Data and Outcomes

Paired reviewers (T.-W. H. and C.-S. L.) extracted data independently using a standardized, pilot-tested form. Reviewers resolved discrepancies by discussion and, when necessary, by adjudication with a third reviewer (Y.-K. T.). The primary outcome was the difference in the change in ADHD symptoms from baseline to the end point between the viloxazine and placebo groups, which was assessed by ADHD Rating Scale–5 (ADHD-RS-5; range, 0-54, with higher scores indicating more severe ADHD symptoms). When data from multiple informants were available, we preferred observer or clinician reports. Intention-to-treat data were prioritized in the analysis to minimize biases from missing data or dropouts. The secondary outcomes were dropout due to AEs, all-cause discontinuation, and the incidence of other AEs, including nausea, headache, somnolence, poor appetite, abdominal pain, and fatigue. Two independent reviewers (T.-W. H. and C.-L. Y.) used the Cochrane Risk of Bias version 2.0 tool (Cochrane Bias Methods Group) for assessing risk of bias (ROB) in the included RCTs. Reviewers resolved discrepancies by discussion and, when necessary, by consulting a senior methodologist (C.-S. L.).

### Statistical Analysis

We used R version 4.2.2 (R Project for Statistical Computing) for all analyses. We calculated mean differences (change in ADHD symptoms) and risk ratios with 95% CIs. The data analysis included 3 steps.

The first step was to pool all effect sizes without considering the dose. This step examined whether viloxazine compared with placebo was associated with the primary and the secondary outcomes without considering the dose or temporal trends. Because fixed-dose RCTs usually have 3 groups or more, the effect sizes in the same study may not be independent. We used random-effects multilevel meta-analyses using the metafor R package. The restricted maximum likelihood method was used to estimate the between-study variance. The multilevel approach can deal with the dependency of effect sizes within a single study. A 3-level meta-analytic model was used. Three sources of variance were modeled, including the sampling variance for the observed effect sizes (level 1), the variance between effect sizes from the same study (level 2), and the variance between the studies (level 3). We used the *I*^2^ statistic to quantify heterogeneity across studies, with values greater than 50% indicating substantial heterogeneity.

The second step was to examine whether viloxazine compared with placebo was associated with the primary and the secondary outcomes in a dose-dependent manner. We conducted 1-stage random-effects dose-response meta-analyses.^21^ We modeled the dose-response associations using restricted cubic splines with 3 knots according to Harrell’s recommended centiles of distribution^22^ (10%, 50%, and 90%). Linear and quadratic models were also examined. Wald χ^2^ tests were performed for testing significance of the dose-response associations, and model comparisons were conducted using likelihood ratio tests and Akaike information criterion. Goodness-of-fit statistics were calculated, and the obtained coefficient of determination (*R*^2^) indicated the variance of the effect size explained by the dose.

The third step was to examine the temporal trends of viloxazine compared with placebo in the primary and the secondary outcomes stratified by different dose. The analyses of this step were similar to those of step 2. Sensitivity analysis was performed taking body weight into account and calculating the daily dosage in milligrams per kilogram when synthesizing the data. All tests of statistical significance were 2-tailed, and *P* < .05 was considered statistically significant.

## Results

### Study Characteristics

Our search resulted in 163 potentially relevant citations (eFigure 1 in Supplement 1). The complete search strategies and reasons for the exclusion of certain studies can be found in eAppendices 1 and 2 in Supplement 1. After removing duplicates, we included 5 double-blind, fixed-dose RCTs.^13,14,15,16,23^ These studies included 1560 participants, with 1084 in the viloxazine group and 476 in the placebo group. The Table shows the characteristics of included studies. Among the participants, 1011 (64.8%) were male, with a mean (SD) age of 10.6 (6.7) years and mean (SD) baseline ADHD-RS-5 scores of 42.3 (4.5) points. There were 2 age groups: 6 to 12 years^14,16,23^ and 12 to 17 years.^13,15^ Two studies^13,23^ had a treatment duration of 6 weeks, 1 study^15^ had a duration of 7 weeks, and 2 studies^16,23^ had a duration of 8 weeks. All participants took the medication throughout the study period. The all-cause discontinuation rates, loss to follow-up rates, and discontinuation due to procedure adherence rates ranged from 14.0%^15^ to 30.1%^23^ for all-cause discontinuation, 3.9%^14^ to 8.3%^16^ for loss to follow-up, and 0.7%^14^ to 1.7%^16^ for discontinuation due to procedure adherence (Table).

**Table.** Characteristics of the Included Studies

Source (trial identifier)	Diagnostic tool	Age group	Study groups	Male participants, No./total sample No. (%)	Mean age, y	Discontinuation, No. (%)			Baseline ADHD-RS score	Treatment duration, wk
						Any	Loss to follow-up	Procedure adherence		
Johnson et al,^23^ 2020 (NCT02633527)	*DSM-5*	6-12 y	Valoxazine 100 mg	27/45 (60)	8.5	11 (24.4)	2 (4.4)	NA	42.4	8
			Valoxazine 200 mg	33/46 (71.7)	9.0	17 (37.0)	5 (10.9)	NA	43.9	
			Valoxazine 300 mg	36/47 (76.6)	9.0	14 (29.8)	5 (10.6)	NA	41.3	
			Valoxazine 400 mg	31/44 (70.5)	9.0	17 (38.6)	4 (9.1)	NA	40.8	
			Placebo	11/24 (45.8)	8.7	3 (12.5)	1 (4.2)	NA	42.4	
Nasser et al,^14^ 2020 (NCT3247530)	*DSM-5*	6-11 y	Valoxazine 100 mg	94/147 (63.9)	8.5	31 (21.1)	9 (6.1)	1 (0.7)	45.0	6
			Valoxazine 200 mg	99/158 (62.7)	8.5	20 (12.7)	6 (3.8)	1 (0.6)	44.0	
			Placebo	97/155 (62.6)	8.5	27 (17.4)	3 (1.9)	1 (0.7)	44.2	
Nasser et al,^15^ 2021 (NCT3247556)	*DSM-5*	12-17 y	Valoxazine 400 mg	66/99 (66.7)	14.0	12 (12.1)	5 (5.1)	0	41.2	7
			Valoxazine 600 mg	71/97 (73.2)	13.7	18 (18.6)	4 (4.1)	1 (1.0)	39.8	
			Placebo	61/96 (63.5)	13.8	12 (12.1)	5 (5.2)	2 (2.1)	38.8	
Nasser et al,^13^ 2021 (NCT3247517)	*DSM-5*	12-17 y	Valoxazine 200 mg	66/94 (70.2)	13.9	20 (21.3)	6 (6.4)	2 (2.1)	39.9	6
			Valoxazine 400 mg	67/103 (65)	14.0	14 (13.6)	4 (3.9)	0	39.4	
			Placebo	58/104 (55.8)	13.8	8 (7.7)	2 (1.9)	0	40.5	
Nasser et al,^16^ 2021 (NCT3247543)	*DSM-5*	6-11 y	Valoxazine 200 mg	74/107 (69.2)	8.5	19 (17.8)	7 (6.5)	3 (2.8)	43.8	8
			Valoxazine 400 mg	59/97 (60.8)	8.4	23 (23.7)	8 (8.2)	1 (1.0)	45.0	
			Placebo	61/97 (62.9)	8.5	17 (17.5)	10 (10.3)	1 (1.0)	43.5	

Abbreviations: ADHD-RS, Attention-Deficit/Hyperactivity Disorder Rating Scale; *DSM-5*, *Diagnostic and Statistical Manual of Mental Disorders, Fifth Edition*; NA, not reported.

### Quality of Evidence

Overall ROB was low in 4 studies^13,14,16,23^ and moderate in 1 study,^15^ with no studies rated as having a high overall ROB. Ratings for individual domains for each study are provided in eFigure 2 in Supplement 1. The proportions of studies with high, some concerns, and low ROB for the individual items of viloxazine trials were as follows: 0 of 5, 4 of 5, and 1 of 5 for randomization; 0 of 5, 1 of 5, and 4 of 5 for deviations from intended interventions; 0 of 5, 0 of 5, and 5 of 5 for missing outcome data; 0 of 5, 0 of 5, and 5 of 5 for measurements of outcomes; and 0 of 5, 0 of 5, and 5 of 5 for selection of reported results.

### Multilevel Meta-Analysis for Main Outcome Without Considering the Dose 

**Figure 1.**
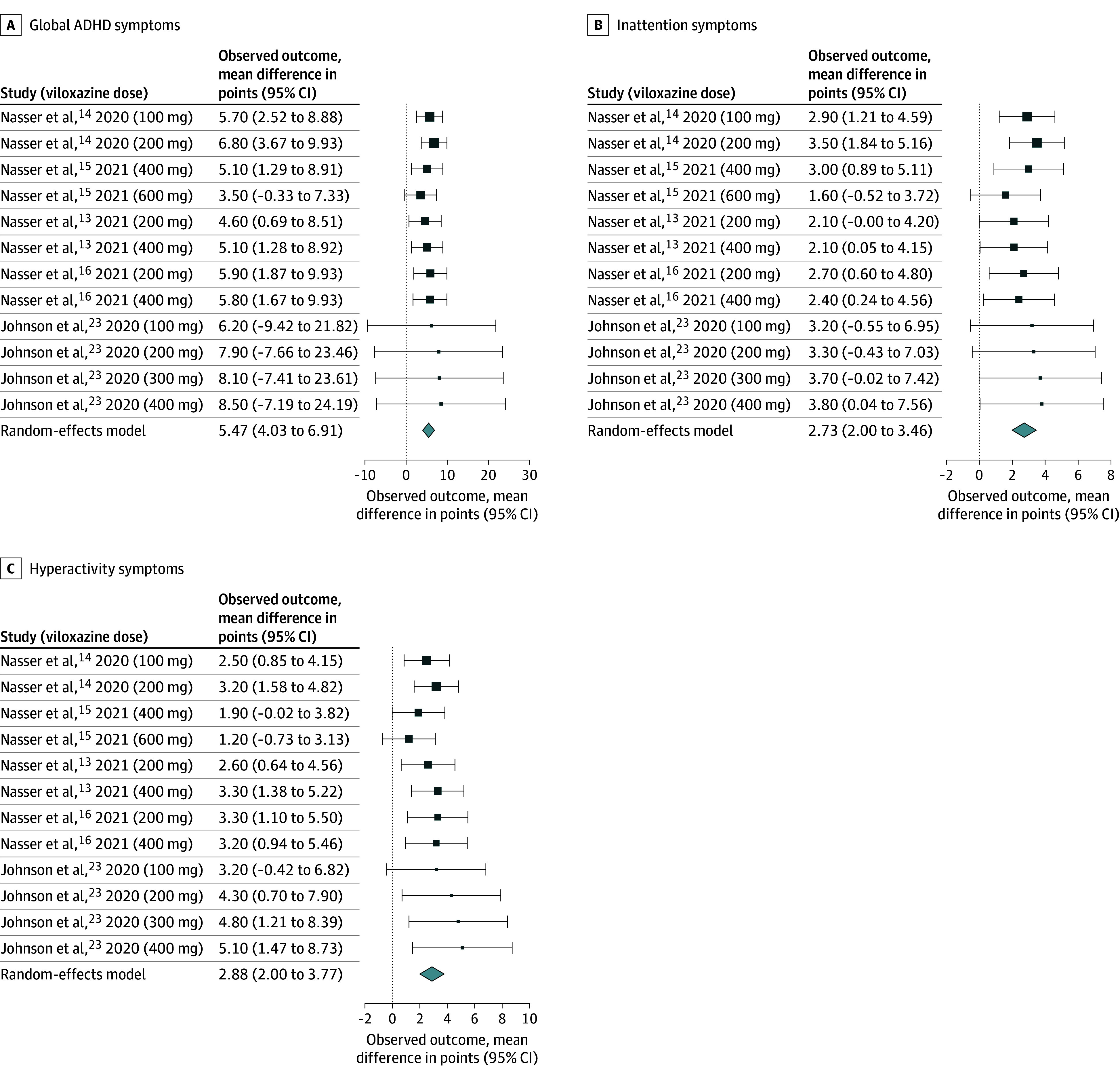
Pairwise Meta-Analysis for Mean Difference of All Attention-Deficit/Hyperactivity (ADHD) Symptoms, Inattention Symptoms, and Hyperactivity Symptoms The sizes of the boxes are proportional to the weight of each study, which is based on the inverse of the variance (precision).

Without considering the dose, viloxazine was associated with significant improvement compared with placebo of global ADHD symptoms measured by ADHD-RS-5 by 5.47 points (95% CI, 4.03-6.91 points; *I*^2^ = 0%) (Figure 1A). Additionally, the improvement was 2.73 points (95% CI, 2.00-3.46 points; *I*^2^ = 0%) for inattention symptoms (Figure 1B) and 2.88 points (95% CI, 2.00-3.77 points; *I*^2^ = 18.35%) for hyperactivity symptoms (Figure 1C).

### Dose-Response Trajectories of ADHD Symptoms

**Figure 2.**
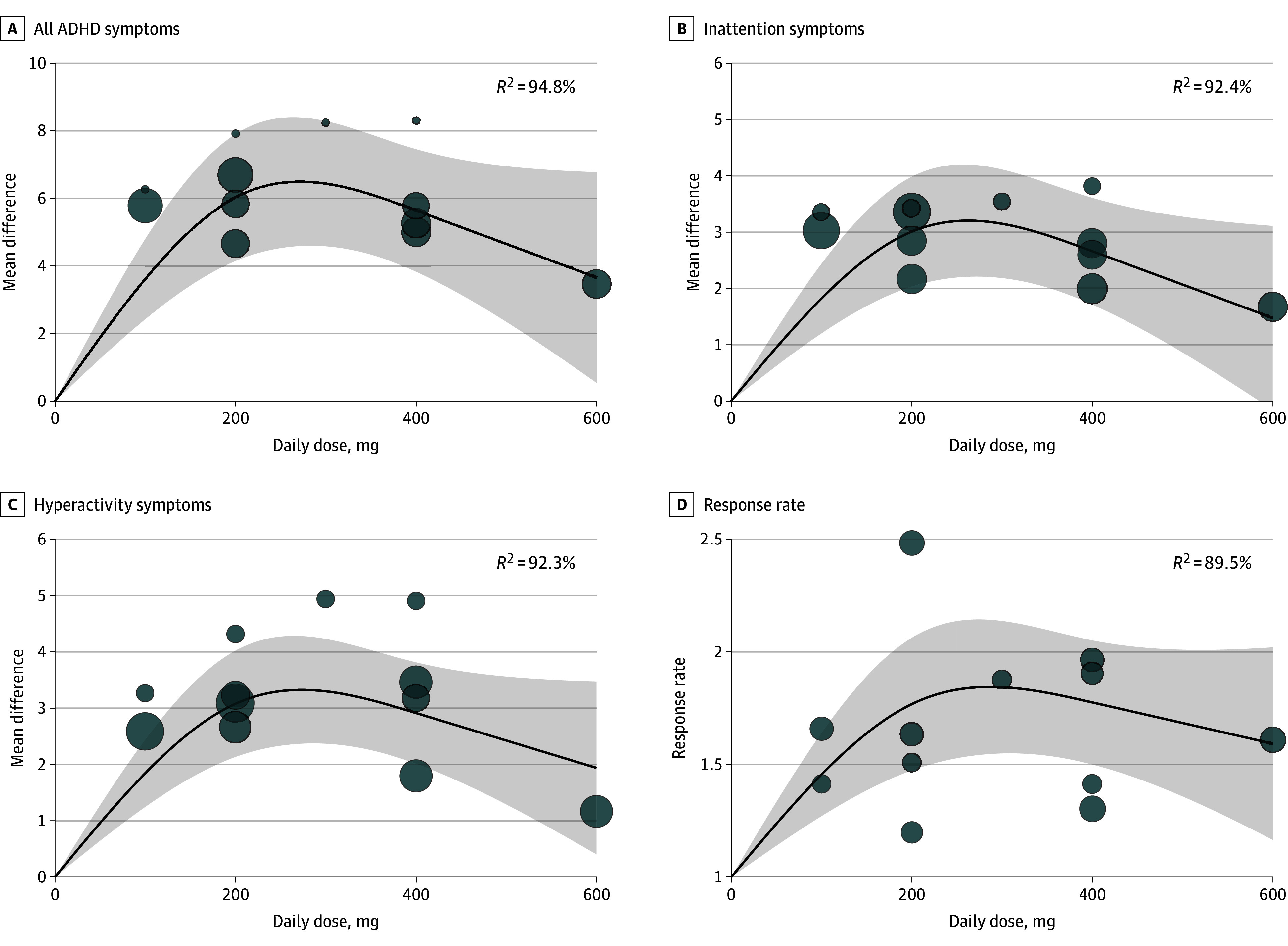
Dose-Response Meta-Analysis for Mean Difference of All Attention-Deficit/Hyperactivity Disorder (ADHD) Symptoms, Inattention Symptoms, Hyperactivity Symptoms, and Response Rate The bubbles are the effect sizes of the original studies, and their sizes are proportional to the inverse of the standard error, with larger bubbles indicating greater precision. The shaded area indicates 95% confidence interval.

**Figure 3.**
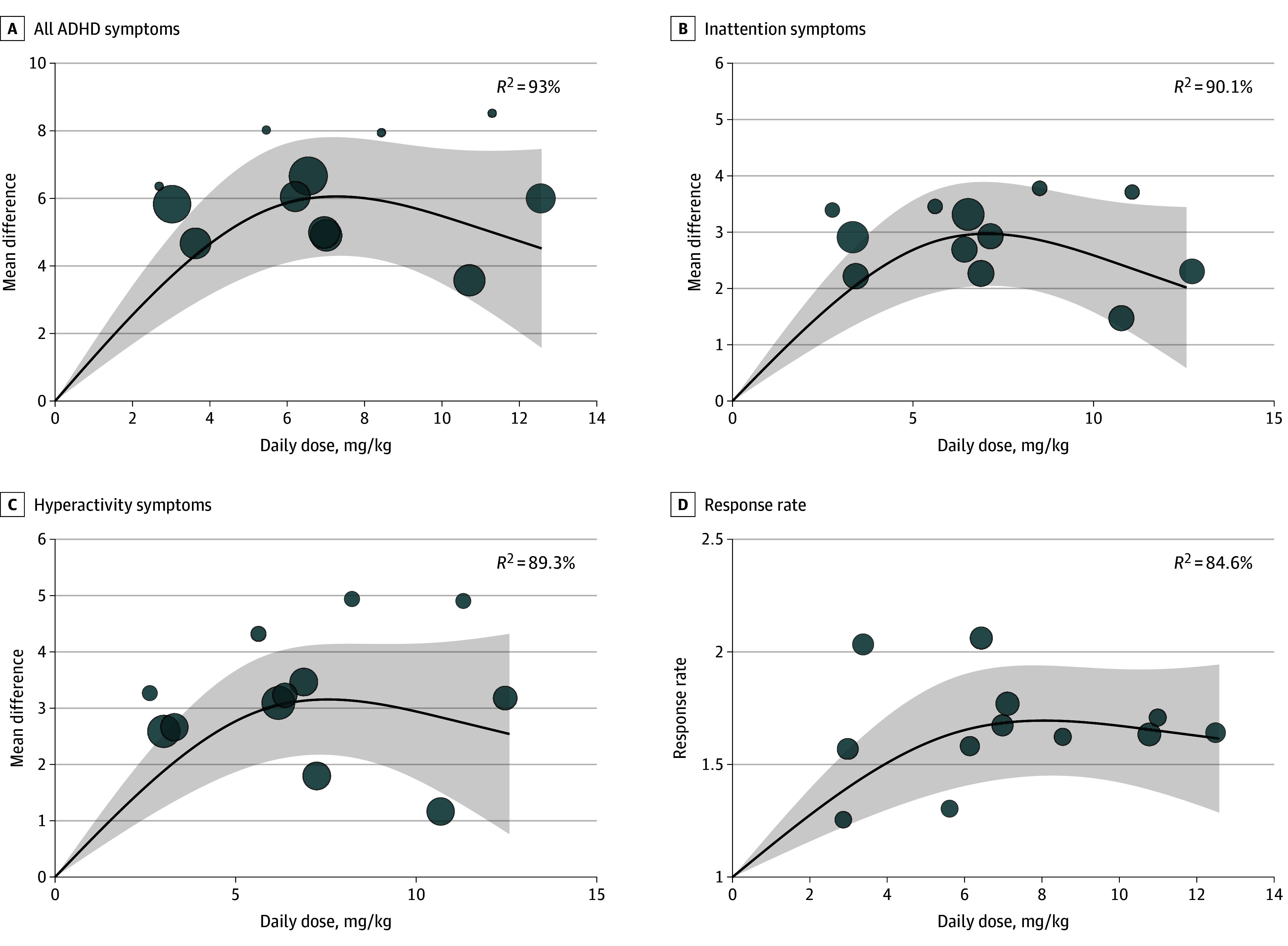
Dose-Response Meta-Analysis When Considering Body Weight for Mean Difference of All Attention-Deficit/Hyperactivity Disorder (ADHD) Symptoms, Inattention Symptoms, Hyperactivity Symptoms, and Response Rate The bubbles are the effect sizes of the original studies, and their sizes are proportional to the inverse of the standard error, with larger bubbles indicating greater precision. The shaded area indicates 95% confidence interval.

Compared with placebo, the dose-response curve of viloxazine on all ADHD symptoms was bell shaped (Figure 2A). The dose with the greatest association with symptom improvement was not 600 mg/d, but rather between 200 mg/d and 400 mg/d. A similar bell-shaped dose-response curve was also observed for inattention symptoms (Figure 2B) and hyperactivity symptoms (Figure 2C). Both dose-response curves of inattention and hyperactivity symptoms showed that the dose with the greatest association with symptom improvement was not 600 mg/d, but rather between 200 mg/d and 400 mg/d. When considering response rate, the dose with the greatest association with symptom improvement was also between 200 mg/d and 400 mg/d (Figure 2D). In the sensitivity analysis that took body weight into account, the dose-response curves for all ADHD symptoms, inattention symptoms, hyperactivity symptoms, and response rate were similar, showing a bell-shaped pattern (Figure 3A-D). The daily dose with the strongest association with symptom improvement was between 6 mg/kg and 8 mg/kg. The supplementary data (eTables 1 and 2 in Supplement 1) show the goodness-of-fit measures for the best-fitting dose-response models.

### Temporal Trends of ADHD Symptoms by Different Viloxazine Doses

**Figure 4.**
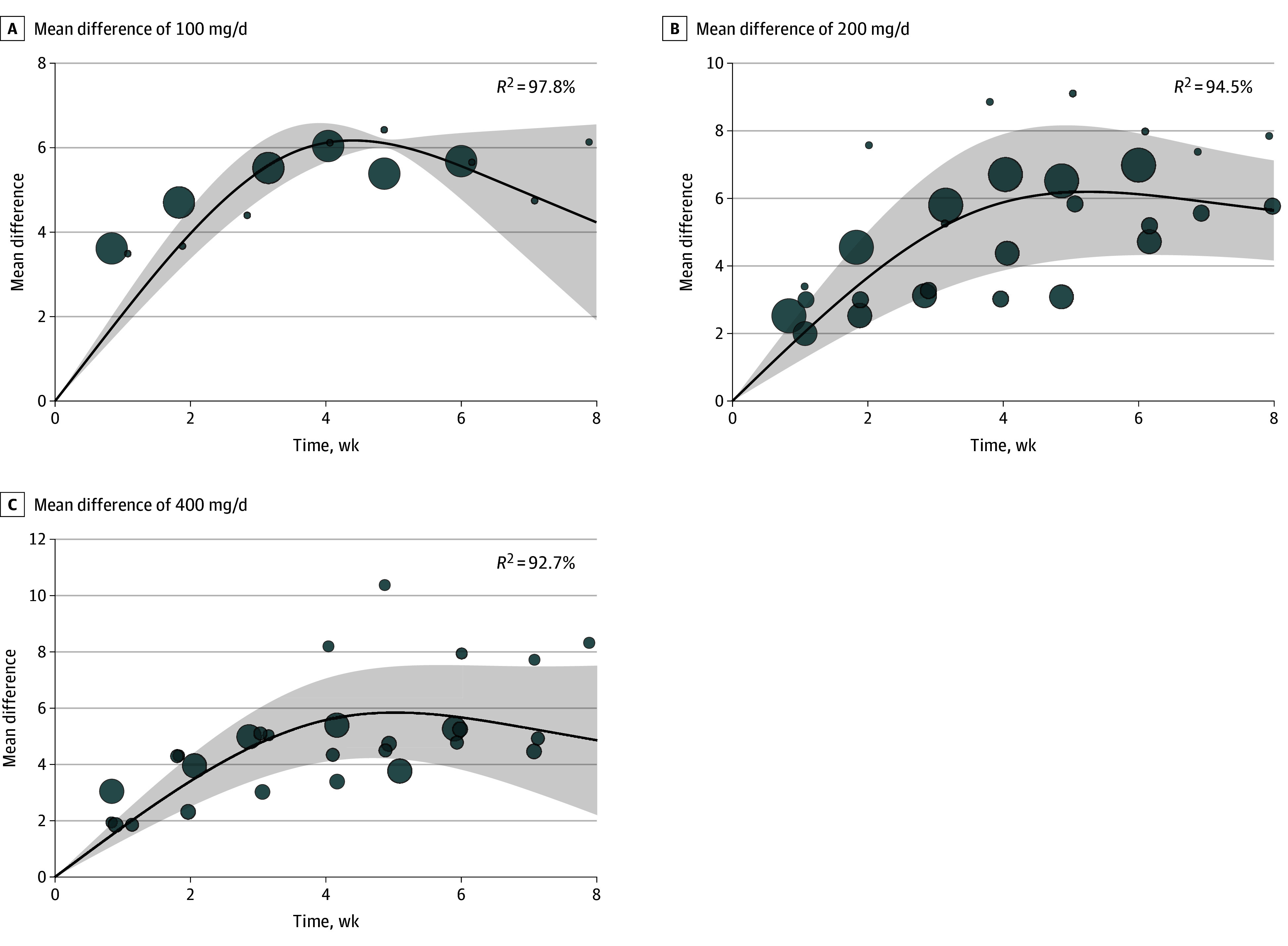
Time-Response Meta-Analysis for Mean Difference of Daily Doses of Viloxazine at 100 mg/d, 200 mg/d, and 400 mg/d The bubbles are the effect sizes of the original studies, and their sizes are proportional to the inverse of the standard error, with larger bubbles indicating greater precision. The shaded area indicates 95% confidence interval.

The temporal trends of all ADHD symptoms on daily doses of 100 mg, 200 mg, and 400 mg were bell-shaped (Figure 4A-C), reaching maximum mean differences after week 4 and tapering off at approximately weeks 4 to 6. The curves for daily dose of 200 mg and 400 mg declined more gradually than that of 100 mg.

### Multilevel Meta-Analysis for Adverse Effects Without Considering the Dose

Without considering the dose, viloxazine compared with placebo was associated with a higher risk of dropout due to AEs (risk ratio [RR], 2.48; 95% CI, 1.26-4.88; *I*^2^ = 0%) (eFigure 9 in Supplement 1). It was also associated with higher risk of the incidence of other AEs, including nausea (RR, 3.06; 95% CI, 1.38-6.77; *I*^2^ = 5.80%) (eFigure 3 in Supplement 1), headache (RR, 2.33; 95% CI, 1.09-5.00; *I*^2^ = 35.81%) (eFigure 4 in Supplement 1), somnolence (RR, 4.33; 95% CI, 2.34-8.01; *I*^2^ = 26.91%) (eFigure 5 in Supplement 1), poor appetite (RR, 7.04; 95% CI, 1.34-36.92; *I*^2^ = 26.49%) (eFigure 6 in Supplement 1), and fatigue (RR, 2.19; 95% CI, 1.02-4.72; *I*^2^ = 9.64%) (eFigure 7 in Supplement 1). However, the risk of all-cause discontinuation and abdominal pain were not significant (eFigures 8 and 9 in Supplement 1). In the viloxazine groups, the overall incidence of dropout due to AEs was 4.15% (45 of 1084 participants). The raw incidences of AEs were presented in eTable 3 in Supplement 1.

### Dose-Response Trajectories of Adverse Effects

There were 4 dose-response curves of AEs that did not show a significant dose-response association: dropout rate, dropout due to AEs, abdominal pain, and fatigue (eFigures 10-13 in Supplement 1). Both the dose-response curves of headache (eFigure 14 in Supplement 1) and poor appetite (eFigure 15 in Supplement 1) showed a linear association. Both the dose-response curves of nausea (eFigure 16 in Supplement 1) and somnolence (eFigure 17 in Supplement 1) were bell-shaped, with higher incidences at doses greater than 200 mg/d.

## Discussion

Our study has several important findings. First, viloxazine was associated with greater efficacy than placebo in treating ADHD symptoms, including both inattention and hyperactivity symptoms. Second, when considering the dose of viloxazine, the dose associated with most symptom improvement was not at 600 mg/d, but rather between 200 mg/d and 400 mg/d. Third, when considering dose and body weight together, the dose associated with the most symptom improvement was between 6 mg/kg and 8 mg/kg. Fourth, the temporal trends showed that the maximum efficacy occurred 4 weeks after taking viloxazine. Fifth, the AEs of poor appetite, headache, somnolence, and nausea had a dose-response association with viloxazine.

To our knowledge, there has not yet been a head-to-head clinical trial comparing the efficacy of viloxazine with current stimulants. Only 1 open-label study with 50 patients with ADHD (35 children)^24^ comparing the efficacy of atomoxetine (25 mg/d to 100 mg/d) and viloxazine (100 mg/d to 600 mg/d) reported that viloxazine extended release had a greater improvement and worked more rapidly in ADHD symptoms than atomoxetine. Furthermore, viloxazine was better tolerated.^24^ A recent meta-analysis^25^ included 87 clinical trials reported that pooling all stimulant and nonstimulant ADHD medications resulted in improvement in ADHD symptom severity (ADHD-RS-5) by 7.35 points. This effectiveness is similar to that of viloxazine, which showed an improvement of 5.47 points.^25^ However, another meta-analysis^26^ only focused on methylphenidate, reporting an improvement of 9.6 points. This effectiveness appears to be higher than that of viloxazine.^26^ Undoubtedly, head-to-head studies are needed to compare the efficacy of viloxazine with other ADHD medications. On the other hand, our study showed that viloxazine was associated with comparable efficacy in both inattention (mean difference, 2.73 points) and hyperactivity (mean difference, 2.88 points) symptoms. This finding contrasts with a meta-analysis^27^ that suggested that atomoxetine was slightly more effective in addressing hyperactivity than inattention symptoms. Additionally, methylphenidate appears to be more effective in treating the inattention subtype compared with the combined subtype.^26^ This difference highlights the clinical value of viloxazine in the management of ADHD.

A dose-response meta-analysis with 65 RCTs^28^ reported that stimulant methylphenidate had a dose-response trend, with higher doses providing better treatment for ADHD symptoms in children and adolescents.^28^ However, the curve demonstrated incrementally less additional improvement beyond 30 mg/d. Another dose-response meta-analysis of 12 RCTs^29^ reported that nonstimulant atomoxetine increased up to a dosage of 1.4 mg/kg, after which it reached a plateau.^29^

Being a nonstimulant, the dose-response curve of viloxazine is similar to that of atomoxetine. In the pharmacokinetics study of viloxazine,^30^ the concentration of viloxazine in the blood is directly proportional to the dosage used. At a dosage of 200 mg, the average concentration in children aged 6 to 11 years is 1.45 µg/mL, and in adolescents aged 12 to 17 years, it is 1.07 µg/mL.^30^ At a dosage of 400 mg, the average concentration in children aged 6 to 11 years is 2.83 µg/mL, and in adolescents aged 12 to 17 years, it is 2.12 µg/mL.^30^ The cutoff point for treatment efficacy in this study was between 200 mg and 400 mg, and therefore, the cutoff point for the drug concentration in the blood might also be around an average concentration of 2.0 µg/mL. Although the 5 trials we included have 2 different age groups (6-11 years and 12-17 years) and the pharmacokinetics study also used age-based grouping, there is a significant correlation between age and weight in the pediatric population. Relative to weight, the effect of age on viloxazine pharmacokinetics can be considered negligible.^30^ Therefore, in the analysis of the dose-response curve, we only considered the parameter of dose and body weight.

In the previously mentioned meta-analysis that included 87 trials,^25^ the treatment durations ranged from 3 to 28 weeks.^25^ After conducting a meta-regression analysis, it was found that the treatment duration did not moderate the efficacy of pharmacological treatment.^25^ In our analysis, we found that the association between viloxazine and effective outcomes reaches a plateau between 4 to 6 weeks, after which there may be a downward trend. The 5 included trials are all fixed-dose RCTs, each with 3 to 5 arms. In the higher-dose arms, the dosage was gradually increased in a stepwise manner. In the group of children aged 6 to 11 years,^14,16,23^ the dosage started at 100 mg/d and increased by 100 mg each week. In the group aged 12 to 17 years,^13,15^ the dosage started at 200 mg and increased by 200 mg each week until reaching the target dose. Therefore, in the time-dependent analysis, the higher-dose arm did not reach the adequate dose during the first 1 to 2 weeks. This could also partially explain why, in the time-dependent analysis, the efficacy plateau for 200 mg/d or 400 mg/d appeared around the fourth week. However, our analysis differs slightly from that of Castells et al.^25^ Their analysis only extracted the time of end point for each trial, whereas we extracted every measurement point throughout the course of each trial. Our approach might better reflect the actual situation. Nevertheless, in clinical practice, an 8-week follow-up is far from sufficient; we need studies with longer follow-up periods to confirm the long-term effects of viloxazine.

Viloxazine was associated with poorer tolerance compared with placebo when focusing on relative RR. When considering the raw incidences, the incidence of AEs appeared to be still low (overall incidence of drop out due to AEs was 4.15% in all viloxazine groups). The most common AEs leading to discontinuation were primarily concentrated in the gastrointestinal tract (including poor appetite and nausea) and neurological symptoms (such as fatigue, somnolence, and headache), which are similar to atomoxetine.^31^ Participants taking viloxazine did not have AEs like stimulants, such as elevated blood pressure, insomnia, risk of dependence, or psychosis. When it was previously used as an antidepressant in adult patients with depression, the more common AEs were asthenia, tremors, constipation, dry mouth, disturbance of micturition, and nausea, which are somewhat different from those in pediatric patients with ADHD.^32^ Generally, the incidence of AEs with viloxazine was relatively low, and many AEs may have a higher risk at doses greater than medium to high levels (ie, >400 mg). However, we need longer follow-up periods to determine the risks associated with long-term use.

### Limitations

Our study still had several limitations. As a new drug, the sample size of viloxazine RCTs remains small. The follow-up duration in clinical trials is limited to 8 weeks, which is insufficient to determine the treatment effects and AEs of continuous use beyond this time. In our analysis, we used study-level weights rather than individual-level weights to calculate the dose per kilogram of viloxazine; however, the distribution of body weight in the included studies did not show skewness. Furthermore, we were unable to assess sex differences due to the lack of separate data. The metabolism of boys and girls may differ, especially after puberty when influenced by sex-specific hormones. We did not perform funnel plots and the Egger test for publication bias because these methods are underpowered and may yield unreliable results when fewer than 10 studies are included. Furthermore, we only analyzed the results of ADHD-RS-5 scale. Other domains, such as cognitive function, executive function, social activity, or risky activity, are all important for patients with ADHD. Moreover, there was only 1 study using 600 mg. We need more studies to evaluate the efficacy and safety of viloxazine using this dose.

## Conclusions

In this meta-analysis in treating children and adolescents with ADHD, we found that viloxazine was associated with more effective outcomes than placebo. The moderate dose (200 mg to 400 mg or 6 mg/kg to 8 mg/kg) may provide an optimal treatment effect. Additionally, viloxazine was well-tolerated, with a low dropout rate and rate of AEs. Nevertheless, more studies with longer follow-up periods are needed to confirm the sustained long-term effects and AEs of viloxazine.

## Notes

Supplement 1. **eFigure 1. **Study Flowchart **eAppendix 1. **Search Strategy **eAppendix 2**. Reasons for Exclusion **eFigure 2**. Risk of Bias Plot of the Included Studies **eTable 1.** Model Fitting for the Dose-Response Association Between the Dose of Viloxazine and the Outcome **eTable 2. **Model Fitting for the Dose-Response (mg/kg) Association Between the Dose of Viloxazine and the Outcome **eFigure 3.** Pairwise Meta-Analysis for Risk Ratio of Nausea **eFigure 4.** Pairwise Meta-Analysis for Risk Ratio of Headache **eFigure 5.** Pairwise Meta-Analysis for Risk Ratio of Somnolence **eFigure 6.** Pairwise Meta-Analysis for Risk Ratio of Poor Appetite **eFigure 7.** Pairwise Meta-Analysis for Risk Ratio of Fatigue **eFigure 8**. Pairwise Meta-Analysis for Risk Ratio of Abdominal Pain **eTable 3**. Raw Incidence of Adverse Effects **eFigure 9**. Pairwise Meta-Analysis for Risk Ratio of Dropout Due to Adverse Events and All-Cause Discontinuation **eFigure 10.** Dose Response Meta-Analysis for Risk Ratio of All-Cause Discontinuation **eFigure 11.** Dose Response Meta-Analysis for Risk Ratio of Dropout Due to Adverse Events **eFigure 12.** Dose Response Meta-Analysis for Risk Ratio of Abdominal Pain **eFigure 13.** Dose Response Meta-Analysis for Risk Ratio of Fatigue **eFigure 14.** Dose Response Meta-Analysis for Risk Ratio of Headache **eFigure 15.** Dose Response Meta-Analysis for Risk Ratio of Poor Appetite **eFigure 16.** Dose Response Meta-Analysis for Risk Ratio of Nausea **eFigure 17.** Dose Response Meta-Analysis for Risk Ratio of Somnolence

Supplement 2.
Data Sharing Statement
