# Supplementary material for: Response Trajectories and Temporal Trends of Viloxazine Treatment for Young People With ADHD: A Meta-Analysis
Source: JAMA Netw Open. 2024 Nov 19;7(11):e2445885. doi: 10.1001/jamanetworkopen.2024.45885 (PMC11577144; doi:10.1001/jamanetworkopen.2024.45885)
Supplement: Supplement 1. — eFigure 1. Study Flowchart eAppendix 1. Search Strategy eAppendix 2. Reasons for Exclusion eFigure 2. Risk of Bias Plot of the Included Studies eTable 1. Model Fitting for the Dose-Response Association Between the Dose of Viloxazine and the Outcome eTable 2. Model Fitting for the Dose-Response (mg/kg) Association Between the Dose of Viloxazine and the Outcome eFigure 3. Pairwise Meta-Analysis for Risk Ratio of Nausea eFigure 4. Pairwise Meta-Analysis for Risk Ratio of Headache eFigure 5. Pairwise Meta-Analysis for Risk Ratio of Somnolence eFigure 6. Pairwise Meta-Analysis for Risk Ratio of Poor Appetite eFigure 7. Pairwise Meta-Analysis for Risk Ratio of Fatigue eFigure 8. Pairwise Meta-Analysis for Risk Ratio of Abdominal Pain eTable 3. Raw Incidence of Adverse Effects eFigure 9. Pairwise Meta-Analysis for Risk Ratio of Dropout Due to Adverse Events and All-Cause Discontinuation eFigure 10. Dose Response Meta-Analysis for Risk Ratio of All-Cause Discontinuation eFigure 11. Dose Response Meta-Analysis for Risk Ratio of Dropout Due to Adverse Events eFigure 12. Dose Response Meta-Analysis for Risk Ratio of Abdominal Pain eFigure 13. Dose Response Meta-Analysis for Risk Ratio of Fatigue eFigure 14. Dose Response Meta-Analysis for Risk Ratio of Headache eFigure 15. Dose Response Meta-Analysis for Risk Ratio of Poor Appetite eFigure 16. Dose Response Meta-Analysis for Risk Ratio of Nausea eFigure 17. Dose Response Meta-Analysis for Risk Ratio of Somnolence [file jamanetwopen-e2445885-s001.pdf]

Supplemental Online Content

Yu C, Kao Y, Thompson T, et al. Response trajectories and temporal trends of viloxazine treatment for young people with ADHD: a meta-analysis. *JAMA Netw Open.* 2024;7(11):e2445885. doi:10.1001/jamanetworkopen.2024.45885

eFigure 1. Study Flowchart

eAppendix 1. Search Strategy

eAppendix 2. Reasons for Exclusion

eFigure 2. Risk of Bias Plot of the Included Studies

eTable 1. Model Fitting for the Dose-Response Association Between the Dose of Viloxazine and the Outcome

eTable 2. Model Fitting for the Dose-Response (mg/kg) Association Between the Dose of Viloxazine and the Outcome

eFigure 3. Pairwise Meta-Analysis for Risk Ratio of Nausea

eFigure 4. Pairwise Meta-Analysis for Risk Ratio of Headache

eFigure 5. Pairwise Meta-Analysis for Risk Ratio of Somnolence

eFigure 6. Pairwise Meta-Analysis for Risk Ratio of Poor Appetite

eFigure 7. Pairwise Meta-Analysis for Risk Ratio of Fatigue

eFigure 8. Pairwise Meta-Analysis for Risk Ratio of Abdominal Pain

eTable 3. Raw Incidence of Adverse Effects

eFigure 9. Pairwise Meta-Analysis for Risk Ratio of Dropout Due to Adverse Events and All-Cause Discontinuation

eFigure 10. Dose Response Meta-Analysis for Risk Ratio of All-Cause Discontinuation

eFigure 11. Dose Response Meta-Analysis for Risk Ratio of Dropout Due to Adverse Events

eFigure 12. Dose Response Meta-Analysis for Risk Ratio of Abdominal Pain

eFigure 13. Dose Response Meta-Analysis for Risk Ratio of Fatigue

eFigure 14. Dose Response Meta-Analysis for Risk Ratio of Headache

eFigure 15. Dose Response Meta-Analysis for Risk Ratio of Poor Appetite

eFigure 16. Dose Response Meta-Analysis for Risk Ratio of Nausea

© 2024 Yu C et al. *JAMA Network Open.*

eFigure 17. Dose Response Meta-Analysis for Risk Ratio of Somnolence

This supplemental material has been provided by the authors to give readers additional information about their work.

© 2024 Yu C et al. *JAMA Network Open.*

eFigure 1. Study Flowchart

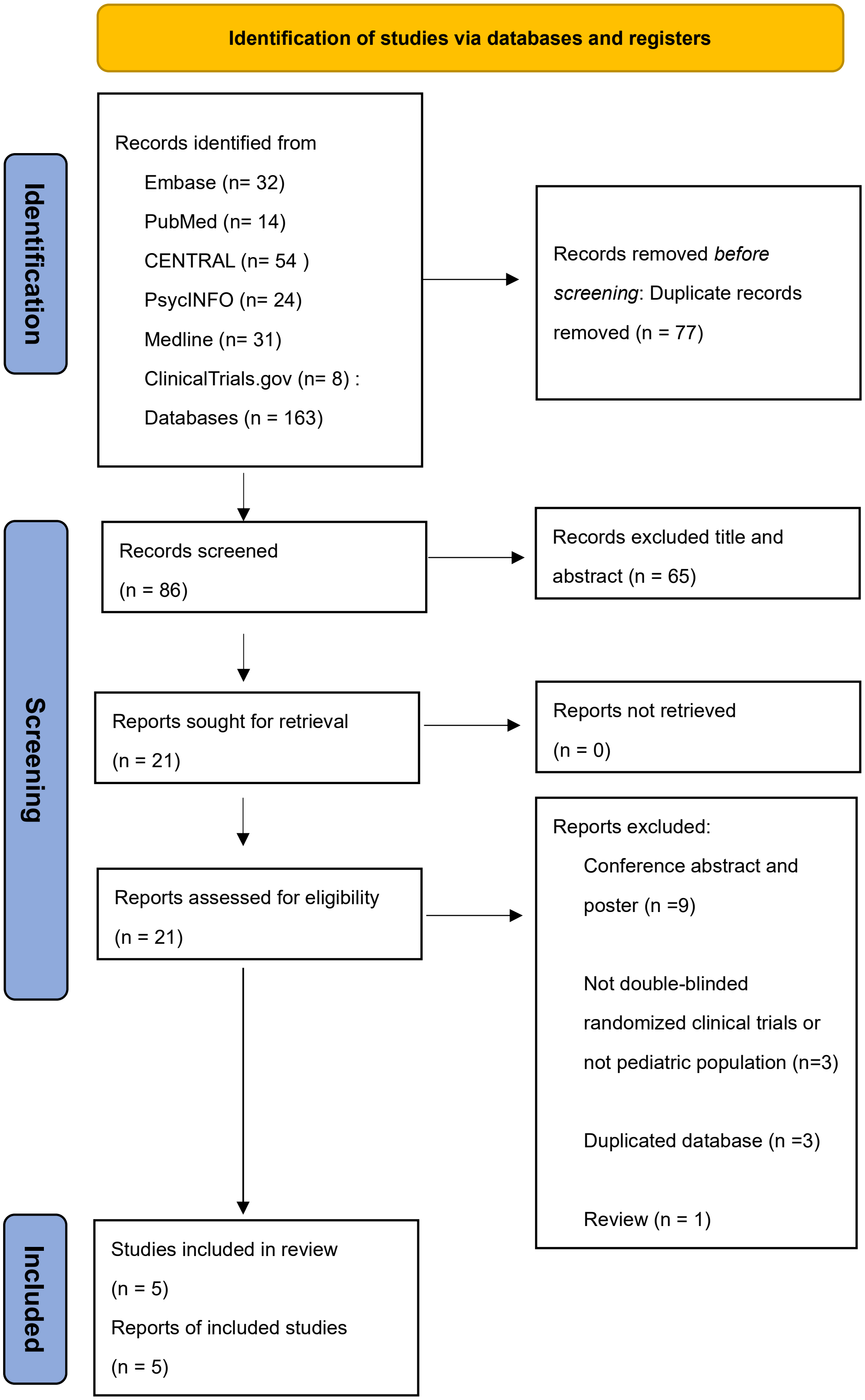

From: Page MJ, McKenzie JE, Bossuyt PM, Boutron I, Hoffmann TC, Mulrow CD, et al. The PRISMA 2020 statement: an updated guideline for reporting systematic reviews. BMJ 2021;372:n71. doi: 10.1136/bmj.n71

© 2024 Yu C et al. *JAMA Network Open.*

eAppendix 1. Search Strategy

Database
**PubMed search strategy**
#1 "viloxazin"[All Fields] OR "viloxazine"[MeSH Terms] OR "viloxazine"[All Fields] (408)<br>#2 "attention deficit disorder with hyperactivity"[MeSH Terms] OR ("attention"[All Fields] AND "deficit"[All Fields] AND "disorder"[All Fields] AND "hyperactivity"[All Fields]) OR "attention deficit disorder with hyperactivity"[All Fields] OR "adhd"[All Fields] (51,100)<br>#3 #1 AND #2 (68)<br>#4 #3 AND (randomizedcontrolledtrial[Filter]) (14)
**Cochrane Central Register of Controlled Trials (CENTRAL) search strategy**
#1 MeSH descriptor: [Viloxazine] explode all trees (69)<br>#2 (viloxazine):ti,ab,kw (Word variations have been searched) (158)<br>#3 MeSH descriptor: [Attention Deficit Disorder with Hyperactivity] explode all trees (3,836)<br>#4 ADHD (6,146)<br>#5 ("Attention Deficit Disorder with Hyperactivity"):ti,ab,kw (3,868)<br>#6 #1 OR #2 (158)<br>#7 #3 OR #4 OR #5 (6,890)<br>#8 #6 AND #7 (54)
**Embase search strategy**
#1. 'viloxazine'/exp OR viloxazine (1,646)<br>#2. 'attention deficit hyperactivity disorder'/exp OR 'attention deficit hyperactivity disorder' OR 'adhd'/exp OR adhd (89,423)<br>#3. #1 AND #2 (147)<br>#4. #3 AND 'randomized controlled trial'/de (32)
**PsycInfo search strategy**
S1 Viloxazine AND ((attention deficit disorder with hyperactivity or adhd)) (24)
**Medline search strategy**
1 Viloxazine/ or Viloxazine.mp. (395)<br>2 Attention Deficit Disorder with Hyperactivity/ or Attention Deficit Disorder with Hyperactivity.mp. or ADHD.mp. (44,956)<br>3 randomized controlled trial.pt. (613,775)<br>4 controlled clinical trial.pt. (95,538)<br>5 randomized.ab. (638,049)<br>6 placebo.ab. (246,320)<br>7 clinical trials as topic.sh. (202,458)

© 2024 Yu C et al. *JAMA Network Open.*

8 randomly.ab. (429,890)
9 trial.ti. (305,586)
10 3 or 4 or 5 or 6 or 7 or 8 or 9 (1,586,859)
11 exp animals/ not humans.sh. (5,225,418)
12 10 not 11 (1,460,560)
13 1 and 2 and 12 (31)
**ClinicalTrials.gov (https://clinicaltrials.gov/)**
Attention Deficit Disorder With Hyperactivity | Viloxazine (8)

CENTRAL: Cochrane Central Register of Controlled Trials

eAppendix 2. Reasons for Exclusion

Not DBRCT, not pediatric population (n=3)

1. A Phase IV, Open-Label, Flexible-Dose Safety Trial Evaluating SPN-812 Administered With Psychostimulants in Children and Adolescents (6 to 17 Years of Age) With Attention-Deficit/Hyperactivity Disorder (ADHD) [Internet]. 2021. Available from: https://clinicaltrials.gov/study/NCT04786990.
2. Nasser A, Hull JT, Chaturvedi SA, Liranso T, Odebo O, Kosheleff AR, et al. A Phase III, Randomized, Double-Blind, Placebo-Controlled Trial Assessing the Efficacy and Safety of Viloxazine Extended-Release Capsules in Adults with Attention-Deficit/Hyperactivity Disorder. CNS drugs. 2022;36(8):897-915.
3. Nct. Evaluation of SPN-812 (Viloxazine Extended-release Capsule) in Adults With ADHD. https://clinicaltrialsgov/show/NCT04016779. 2019.

Duplicated data (n=3)

1. Nasser A, Hull JT, Chowdhry FA, Adewole T, Liranso T, Marcus R. Phase 3, randomized, double-blind, placebo-controlled studies evaluating efficacy and safety of extended-release viloxazine (SPN-812) for pediatric ADHD: update on the second adolescent study (p304). Neurotherapeutics. 2019;16(3):915.
2. Nct. Efficacy and Safety of SPN-812 (Viloxazine Extended-release Capsule) in Children With ADHD. https://clinicaltrialsgov/show/NCT02633527. 2015.
3. Nasser A, Hull JT, Liranso T, Busse GD, Melyan Z, Childress AC, et al. The effect of viloxazine extended-release capsules on functional impairments associated with attention-deficit/hyperactivity disorder (ADHD) in children and adolescents in four phase 3 placebo-controlled trials. Neuropsychiatric disease and treatment. 2021;17:1751-62.

Conference abstract or Poster (n=9)

1. Findling RL, Newcorn JH, Cutler AJ, Childress AC, Hull JT, Qin P, et al. 5.13 Evaluation of the Efficacy of Viloxazine ER in ADHD Inattentive and Combined Presentations. Journal of the American Academy of Child and Adolescent Psychiatry. 2023;62(10):S262-S3.
2. Johnson JK, Saylor K, Brittain ST, Tulloch G, Liranso T. A double-blind, placebo-controlled, dose-ranging study of extended-release viloxazine (SPN-812 ER) in children with attention-deficit/hyperactivity disorder (ADHD). Journal of the American Academy of Child and Adolescent Psychiatry. 2017;56(10):S283-S4.
3. Nasser A, Busse GD, Hull J, Chowdhry F, Adewole T, Liranso T, et al. 1.70 A PHASE 3, RANDOMIZED, DOUBLE-BLIND, PLACEBO-CONTROLLED STUDY (P301) ASSESSING THE EFFICACY AND SAFETY OF SPN-812 (EXTENDED-RELEASE VILOXAZINE) 100 AND 200MG FOR THE TREATMENT OF ADHD IN CHILDREN. Journal of the American Academy of Child and Adolescent Psychiatry. 2019;58(10):S169-S70.
4. Nasser A, Hull JT, Chaturvedi S, Adewole T, Liranso T, Busse G, et al. 5.31 THE EFFICACY AND SAFETY OF SPN-812 (VILOXAZINE EXTENDED-RELEASE) FOR THE TREATMENT OF ADHD IN CHILDREN AND ADOLESCENTS. Journal of the American Academy of Child and Adolescent Psychiatry. 2020;59(10):S159.
5. Nasser A, Hull JT, Chowdhry FA, Adewole T, Liranso T, Marcus R. Extended-release viloxazine (SPN-812) for the treatment of attention-deficit/hyperactivity disorder (ADHD) in adolescents: topline results of a phase 3, randomized, double-blind, placebo-controlled study (p302). Neurotherapeutics. 2019;16(3):914-5.

© 2024 Yu C et al. *JAMA Network Open.*

6. Nasser A, Hull JT, Chowdhry FA, Adewole T, Liranso T, Schwabe S. 112 A Phase 3, Randomized, Double-Blind, Placebo-Controlled Study (P302): efficacy and Safety of Extended-Release Viloxazine in Adolescents with ADHD. CNS spectrums. 2020;25(2):272-3.
7. Nasser A, Hull JT, Chowdhry FA, Adewole T, Liranso T, Schwabe S. 113 Phase 3, Randomized, Double-Blind, Placebo-Controlled Study (P303) Assessing Efficacy and Safety of Extended-Release Viloxazine in Children with ADHD. CNS spectrums. 2020;25(2):273-4.
8. Nasser A, Johnson JK, Adewole T, Liranso T, Marcus R. Phase 3 randomized, double-blind, placebo-controlled studies evaluating efficacy and safety of extended-release viloxazine for pediatric ADHD. CNS spectrums. 2019;24(1):177-8.
9. Nasser A, Kosheleff AR, Hull JT, Liranso T, Qin P, Busse GD, et al. Evaluating the likelihood to be helped or harmed after treatment with viloxazine extended-release in children and adolescents with attention-deficit/hyperactivity disorder. International Journal of Clinical Practice. 2021;75(8).

Review (n=1)

1. Raible H, D'Souza MS. Extended-Release Viloxazine for the Treatment of Attention-Deficit Hyperactivity Disorder in School-Age Children and Adolescents. Annals of pharmacotherapy. 2023;57(12):1436-48.

© 2024 Yu C et al. *JAMA Network Open.*

eFigure 2. Risk of Bias Plot of the Included Studies

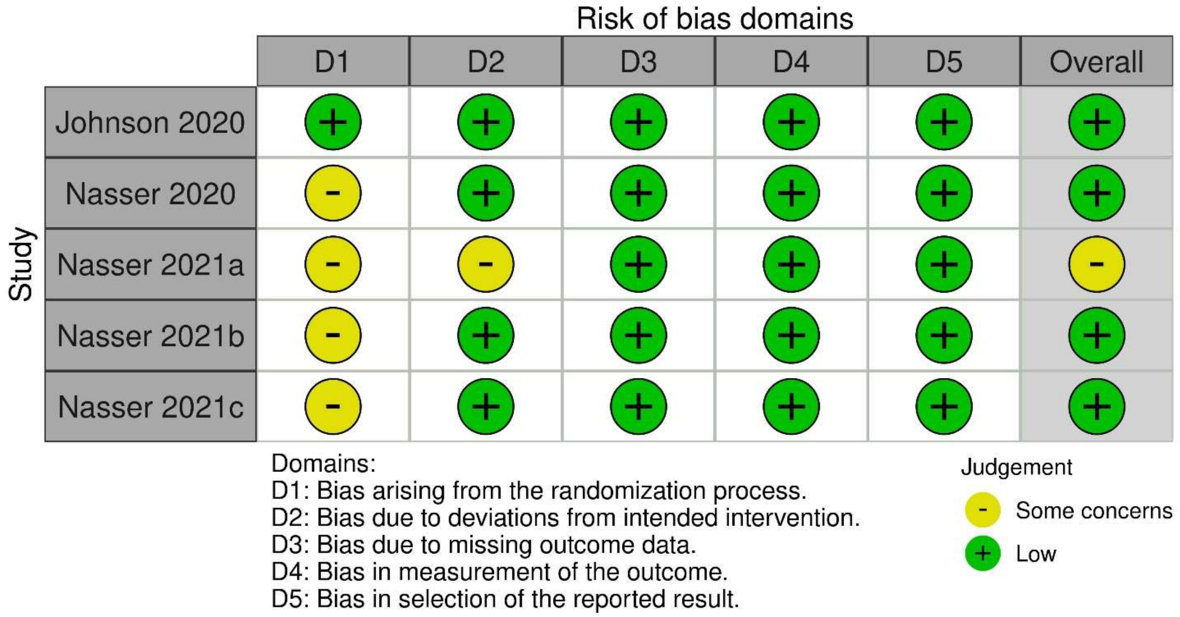

© 2024 Yu C et al. *JAMA Network Open.*

eTable 1. Model Fitting for the Dose-Response Association Between the Dose of Viloxazine and the Outcome

Outcome	Dose-response association	Best model fitting (linear vs quadratic vs spline)[a]	R^2 (Goodness-of-fit)
All ADHD symptoms*	p<0.001	Spline, p<0.001	0.948
Inattention symptoms*	p<0.001	Spline, p<0.001	0.924
Hyperactivity symptoms*	p<0.001	Spline, p<0.001	0.923
Response rate*	p<0.001	Spline, p<0.001	0.923
Response trajectory of 100 mg/d*	p<0.001	Spline, p<0.001	0.978
Response trajectory of 200 mg/d*	p<0.001	Spline, p<0.001	0.945
Response trajectory of 400 mg/d*	p<0.001	Spline, p<0.001	0.927
Dropout	p=0.05	Linear, p=0.05	0.194
Dropout due to adverse effect	p=0.182	Linear, p=0.18	0.156
Nausea*	p<0.001	Spline, p=0.01	0.642
Headache*	p=0.040	Linear, p=0.04	0.269
Somnolence*	p=0.029	Spline, p=0.03	0.709
Poor appetite*	p=0.037	Linear, p=0.04	0.662
Abdominal pain	p=0.916	Linear, p=0.92	0.110
Fatigue	p=0.050	Linear, p=0.05	0.585

[a] spline is restricted cubic spline with three knots (0.1, 0.5, 0.9)

*: statistically significant dose-response association between the dose of viloxazine and the outcome

Abbreviations: ADHD=attention deficit/hyperactivity

© 2024 Yu C et al. *JAMA Network Open.*

eTable 2. Model Fitting for the Dose-Response (mg/kg) Association Between the Dose of Viloxazine and the Outcome

Outcome	Dose-response association	Best model fitting (linear vs quadratic vs spline)[a]	R^2 (Goodness-of-fit)
All ADHD symptoms*	p<0.001	Spline, p<0.001	0.930
Inattention symptoms*	p<0.001	Spline, p<0.001	0.901
Hyperactivity symptoms*	p<0.001	Spline, p<0.001	0.893
Response rate*	p<0.001	Spline, p=0.004	0.846

[a] spline is restricted cubic spline with three knots (0.1, 0.5, 0.9)

*: statistically significant dose-response association between the dose of viloxazine and the outcome

Abbreviations: ADHD=attention deficit/hyperactivity

© 2024 Yu C et al. *JAMA Network Open.*

eFigure 3. Pairwise Meta-Analysis for Risk Ratio of Nausea

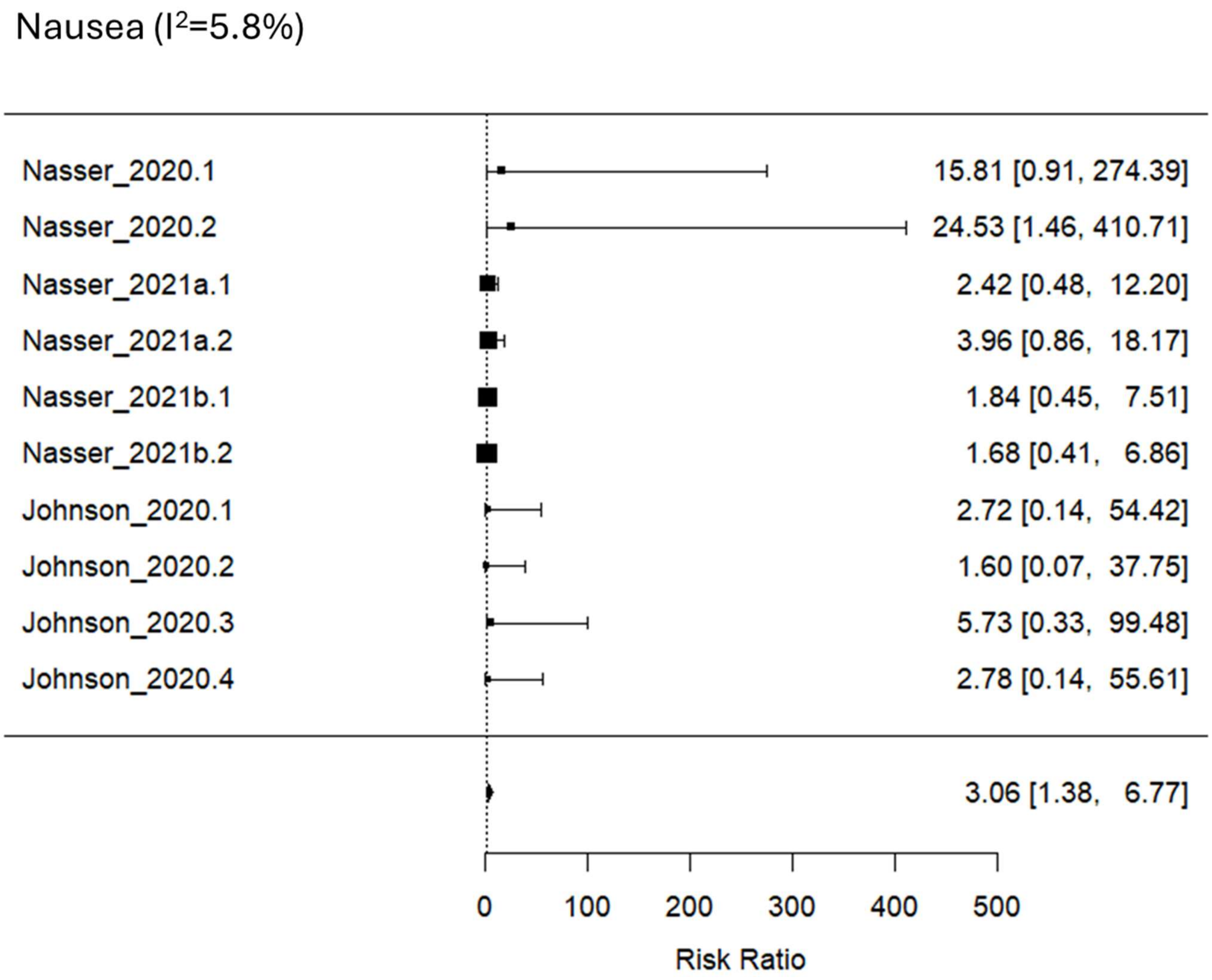

© 2024 Yu C et al. *JAMA Network Open.*

eFigure 4. Pairwise Meta-Analysis for Risk Ratio of Headache

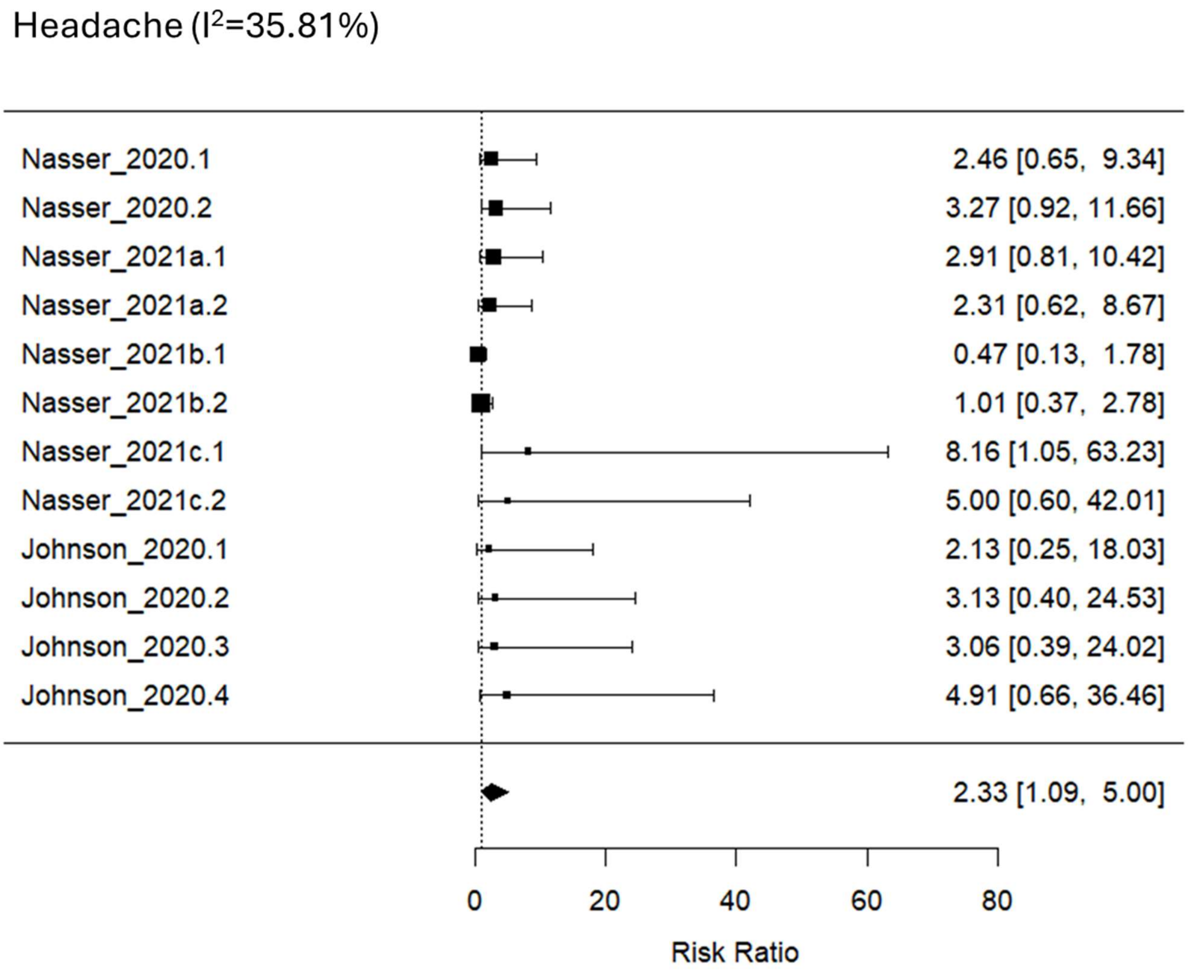

© 2024 Yu C et al. *JAMA Network Open.*

eFigure 5. Pairwise Meta-Analysis for Risk Ratio of Somnolence

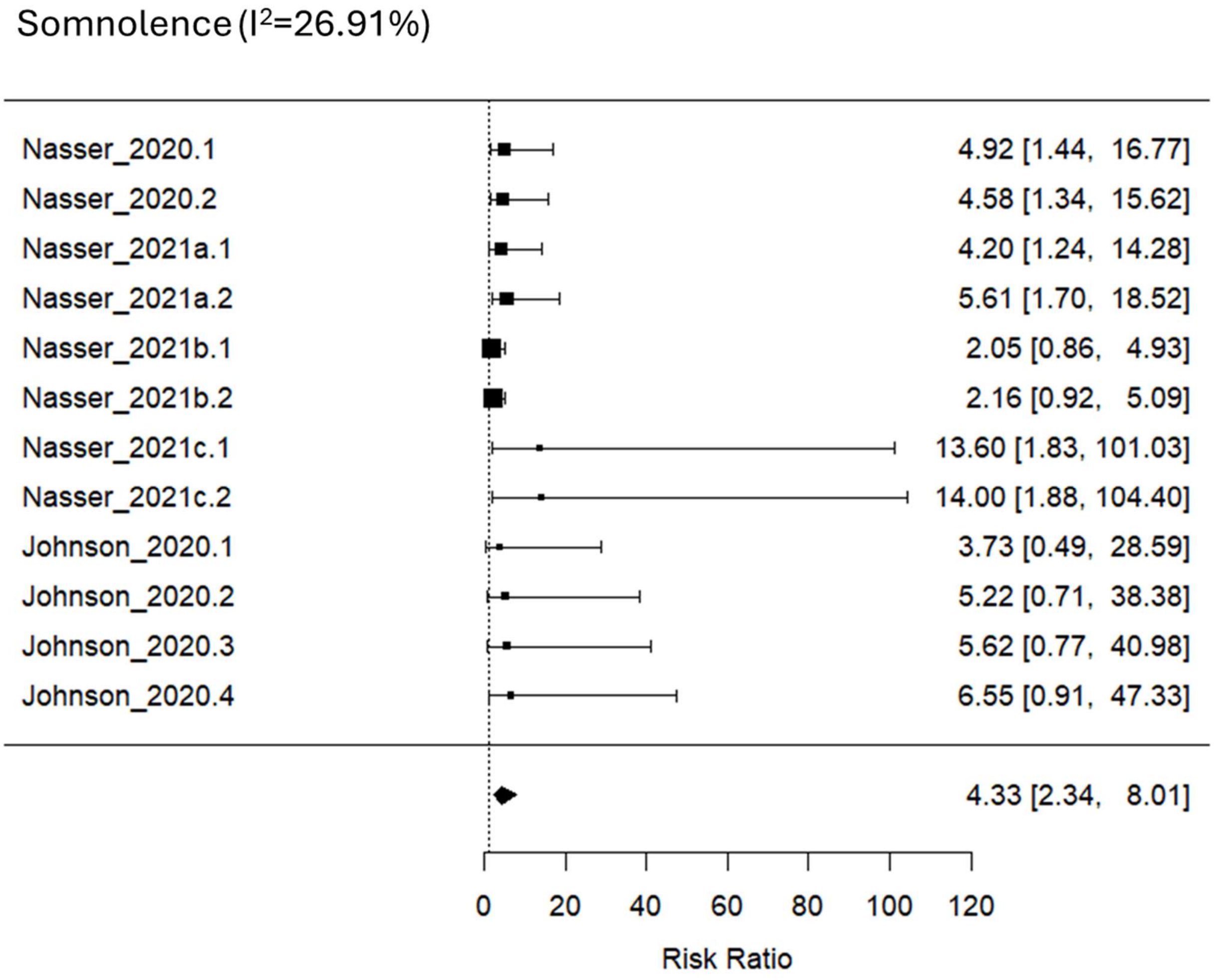

© 2024 Yu C et al. *JAMA Network Open.*

eFigure 6. Pairwise Meta-Analysis for Risk Ratio of Poor Appetite

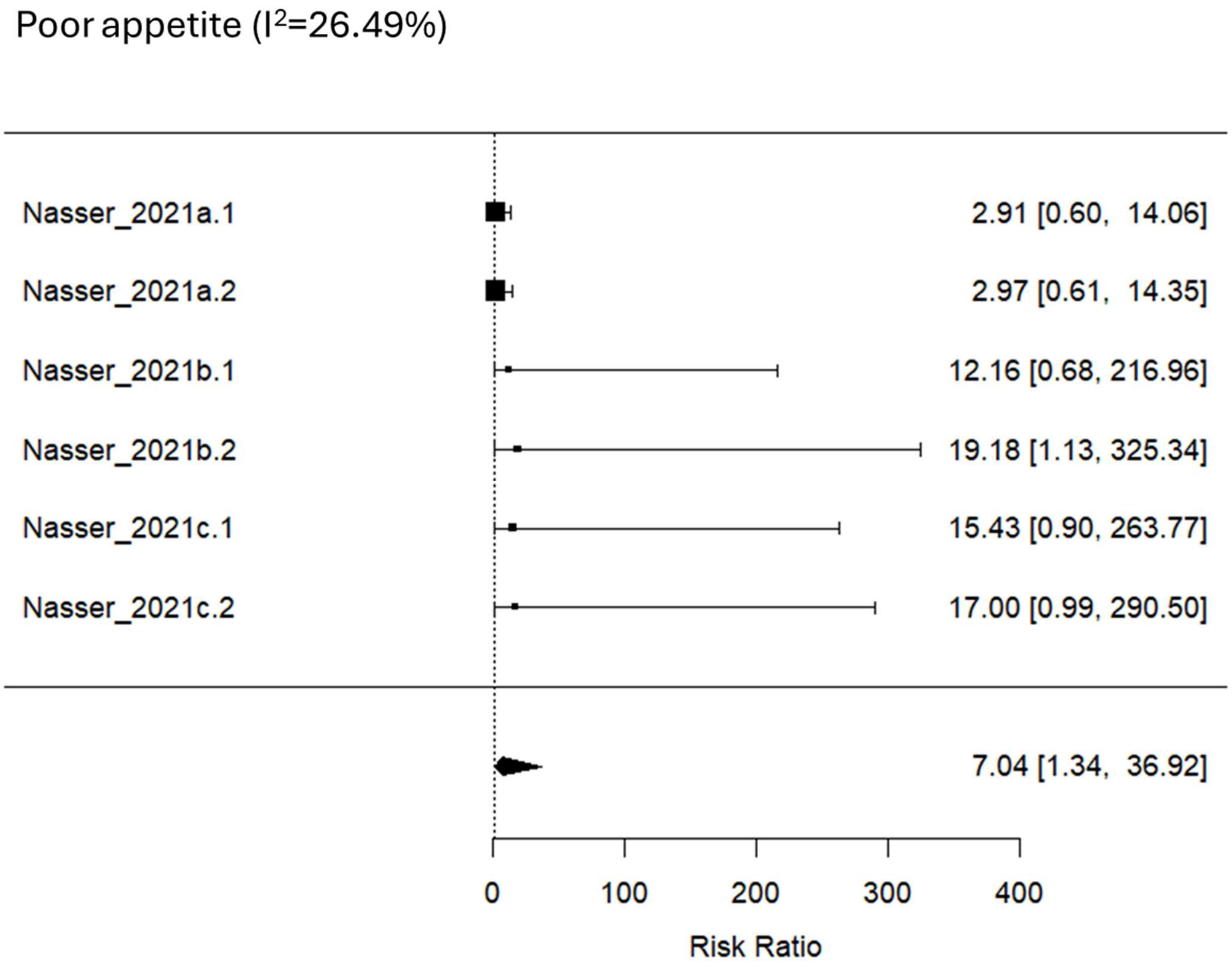

© 2024 Yu C et al. *JAMA Network Open.*

eFigure 7. Pairwise Meta-Analysis for Risk Ratio of Fatigue

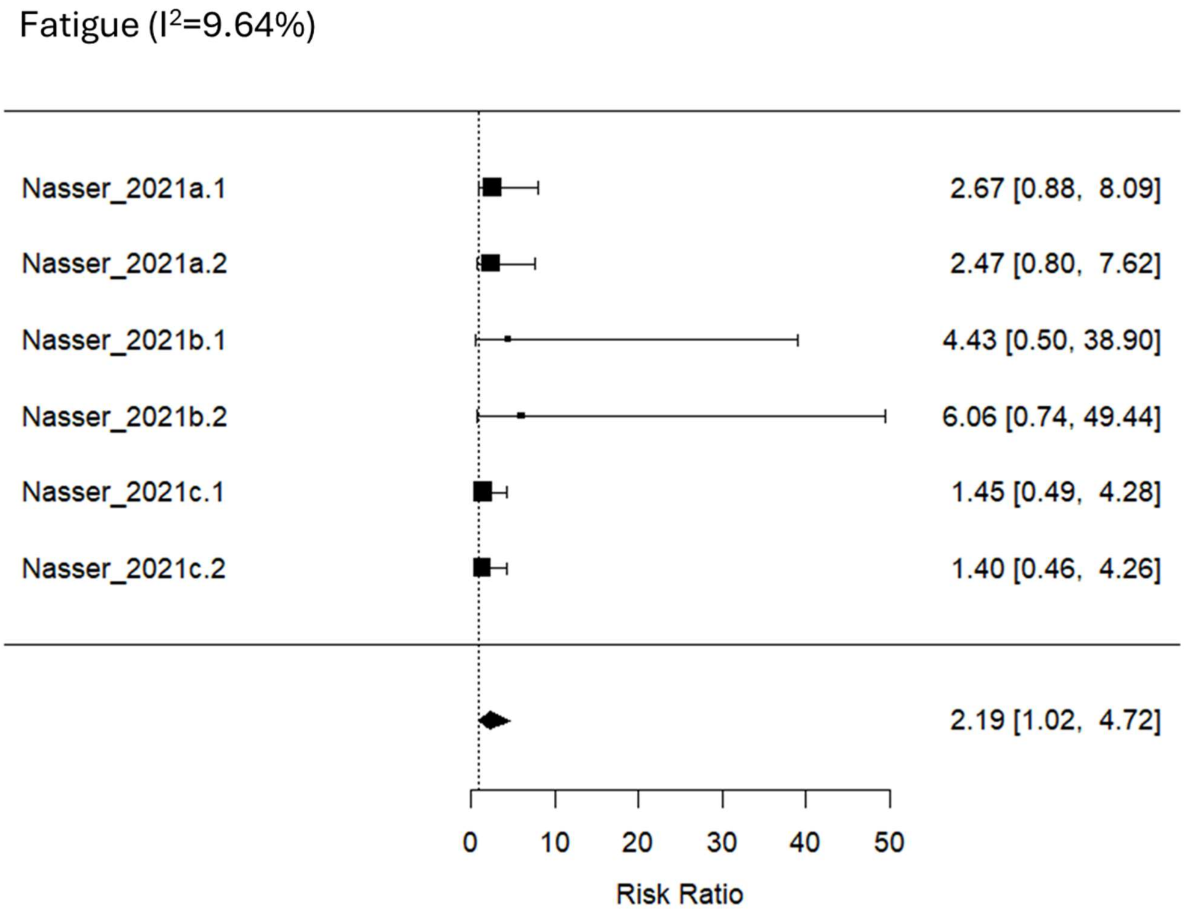

© 2024 Yu C et al. *JAMA Network Open.*

eFigure 8. Pairwise Meta-Analysis for Risk Ratio of Abdominal Pain

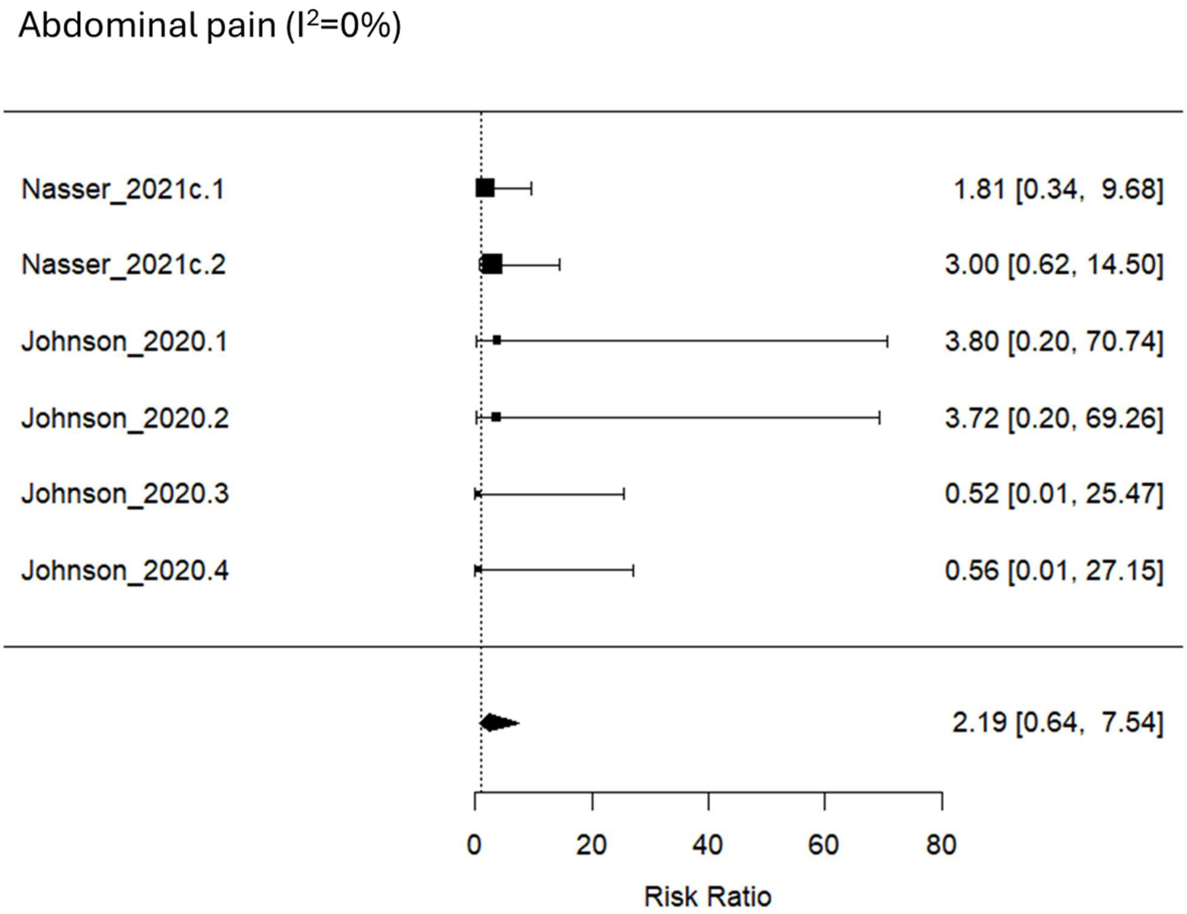

eTable 3. Raw Incidence of Adverse Effects

Study	Intervention arms	sample size	Drop out	Drop out due to AE	Decreased appetite	Abdominal pain	Nausea	Headache	Somnolence	Fatigue
Nasser 2020	Viloxazine 100mg	147	31	5			7	7	14	
	Viloxazine 200mg	158	20	2			12	10	14	
	Placebo	155	27	2			0	3	3	
Nasser 2021a	Viloxazine 400mg	99	12	4	6		5	9	13	11
	Viloxazine 600mg	97	18	5	6		8	7	17	10
	Placebo	96	12	1	2		2	3	3	4
Nasser 2021b	Viloxazine 200mg	94	20	4	5		5	3	13	4
	Viloxazine 400mg	103	14	2	9		5	7	15	6
	Placebo	104	8	0	0		3	7	7	1
Nasser 2021c	Viloxazine 200mg	107	19	6	8	4		9	15	8
	Viloxazine 400mg	97	23	4	8	6		5	14	7
	Placebo	97	17	3	0	2		1	1	5
Johnson 2020	Viloxazine 100mg	45	11	4		3	2	4	7	2
	Viloxazine 200mg	46	17	3		3	1	6	10	2
	Viloxazine 300mg	47	14	1		0	5	6	11	1
	Viloxazine 400mg	44	17	5		0	2	9	12	5
	Placebo	24	3	0		0	0	1	1	0

© 2024 Yu C et al. *JAMA Network Open.*

eFigure 9. Pairwise Meta-Analysis for Risk Ratio of Dropout Due to Adverse Events and All-Cause Discontinuation

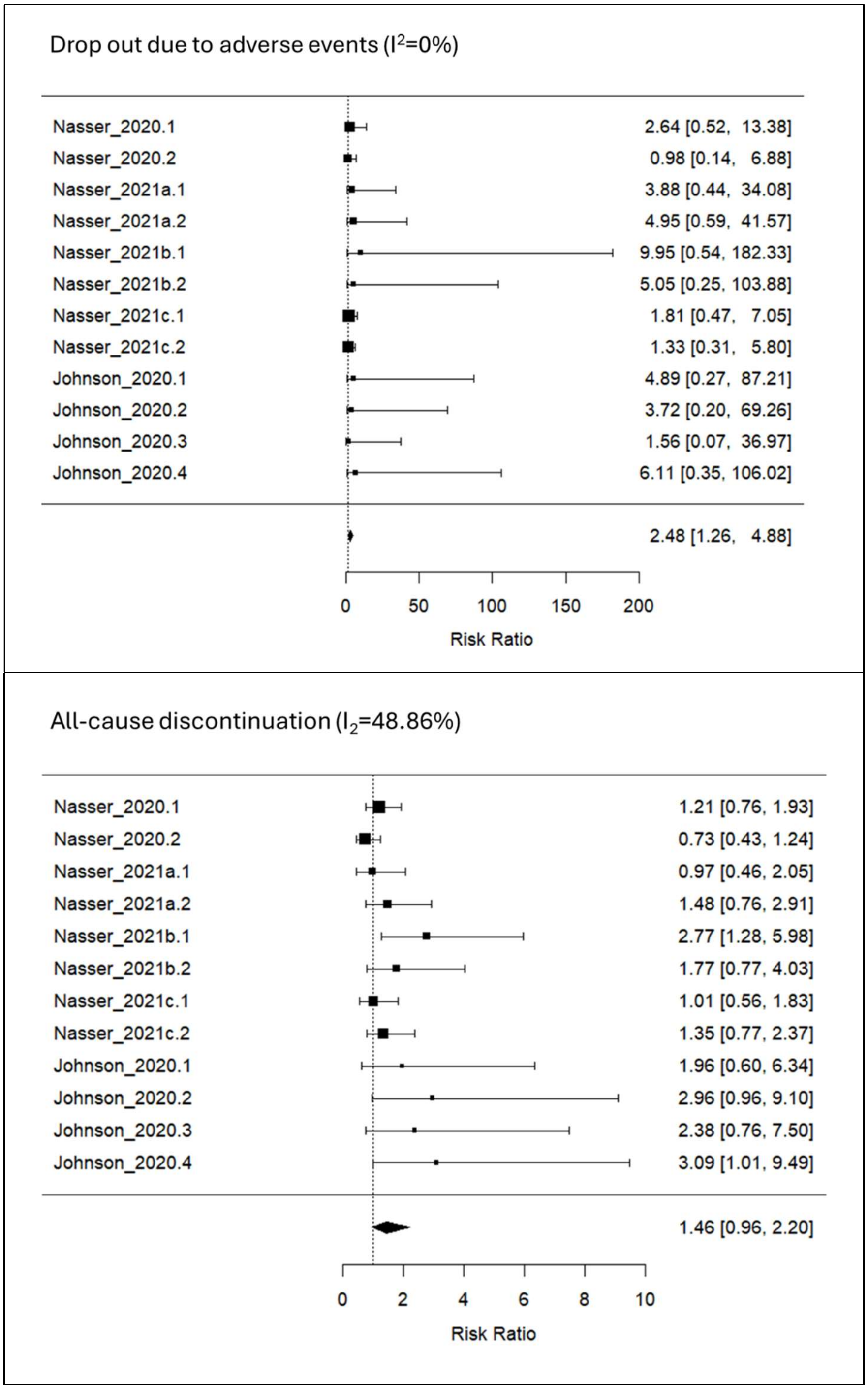

Nasser_2020.1/2 = 100/200mg; Nasser_2021a.1/2 =400/600mg; Nasser_2021b. 1/2= 200/400mg; Nasser_2021c. 1/2= 200/400mg; Johnson_2020.1/2/3/4= 100/200/300/400mg.

© 2024 Yu C et al. *JAMA Network Open.*

eFigure 10. Dose Response Meta-Analysis for Risk Ratio of All-Cause Discontinuation

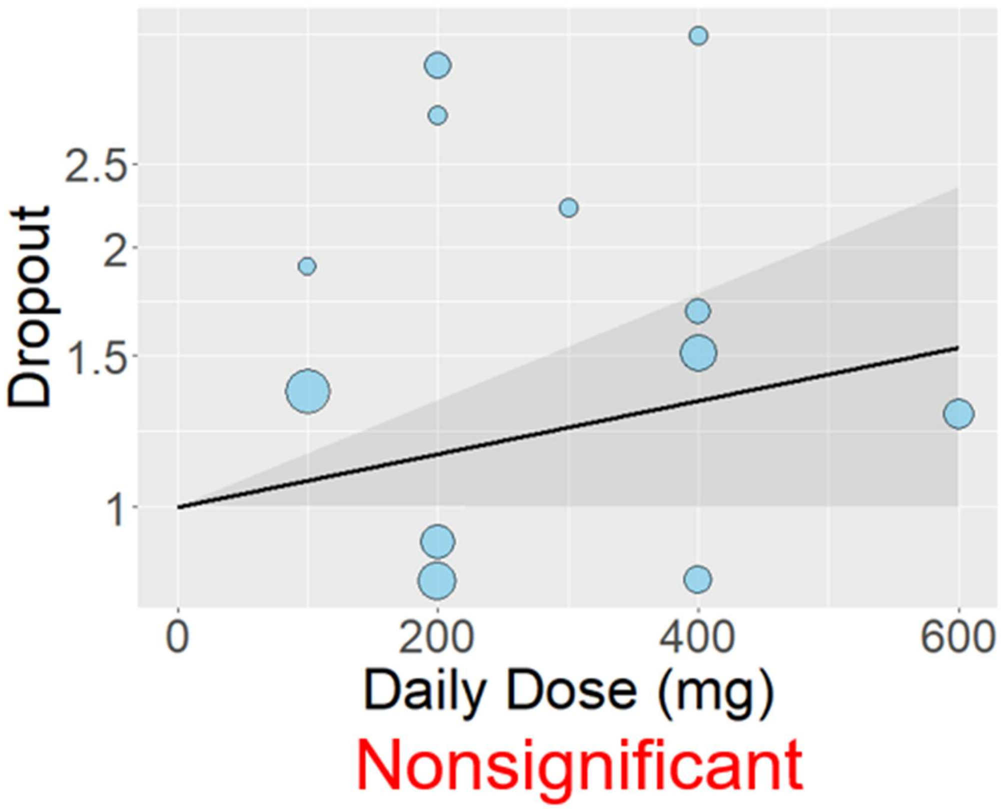

eFigure 11. Dose Response Meta-Analysis for Risk Ratio of Dropout Due to Adverse Events

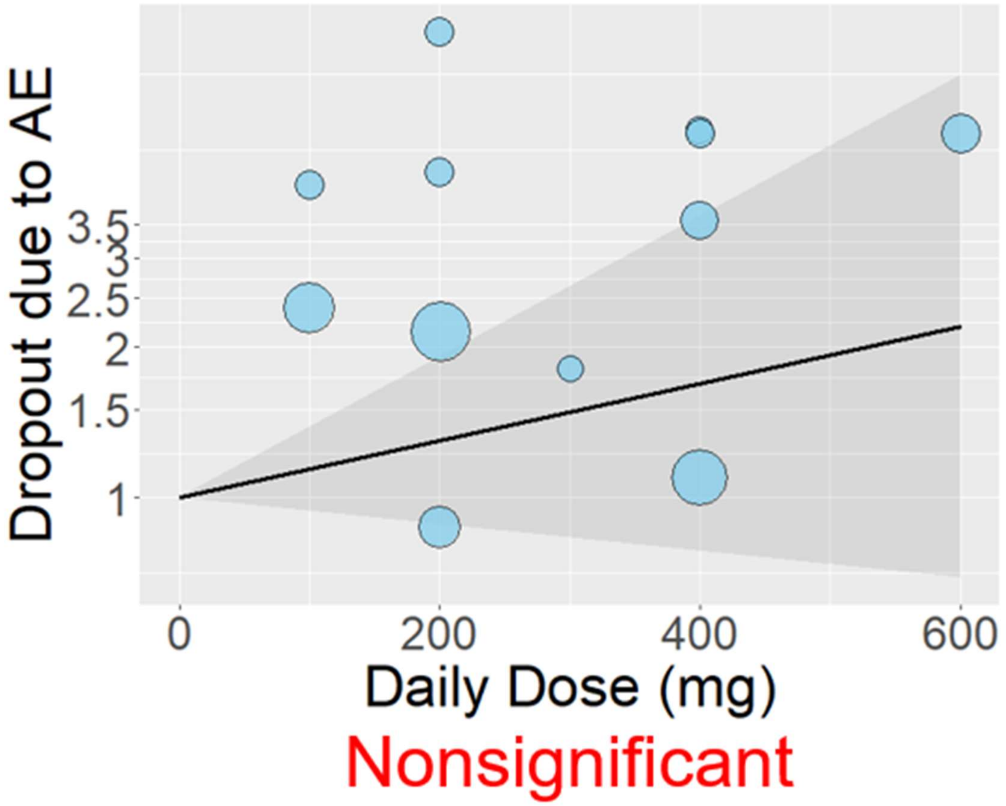

© 2024 Yu C et al. *JAMA Network Open.*

eFigure 12. Dose Response Meta-Analysis for Risk Ratio of Abdominal Pain

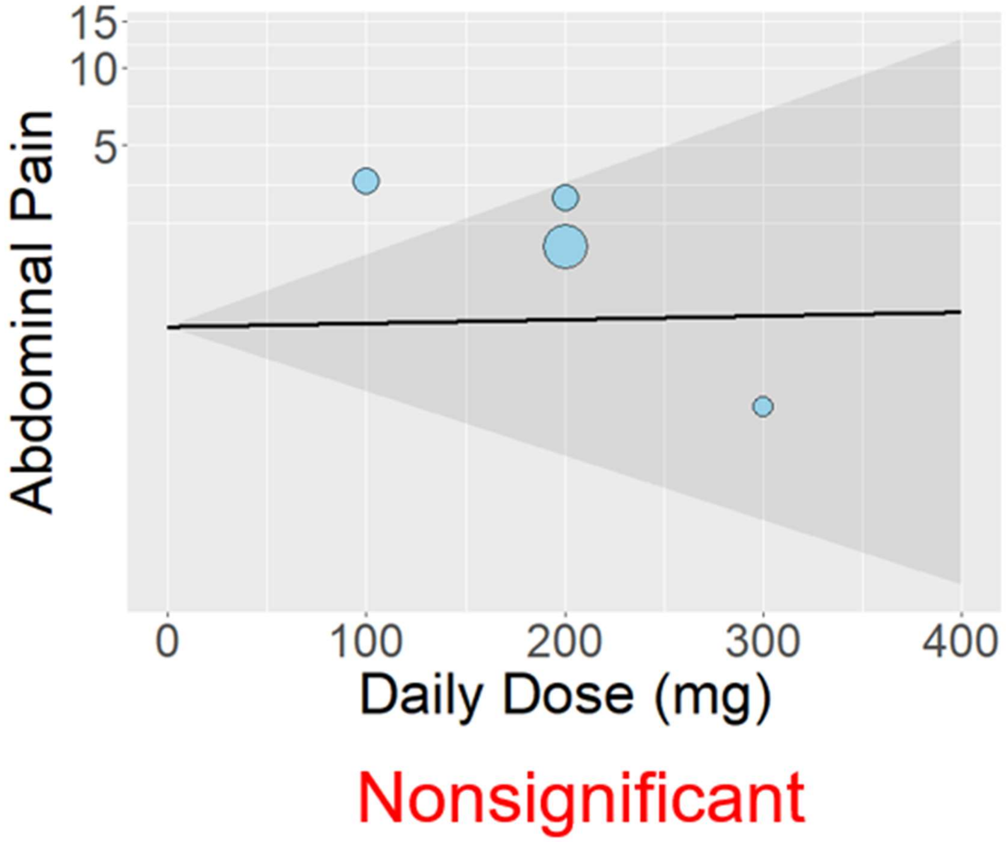

© 2024 Yu C et al. *JAMA Network Open.*

eFigure 13. Dose Response Meta-Analysis for Risk Ratio of Fatigue

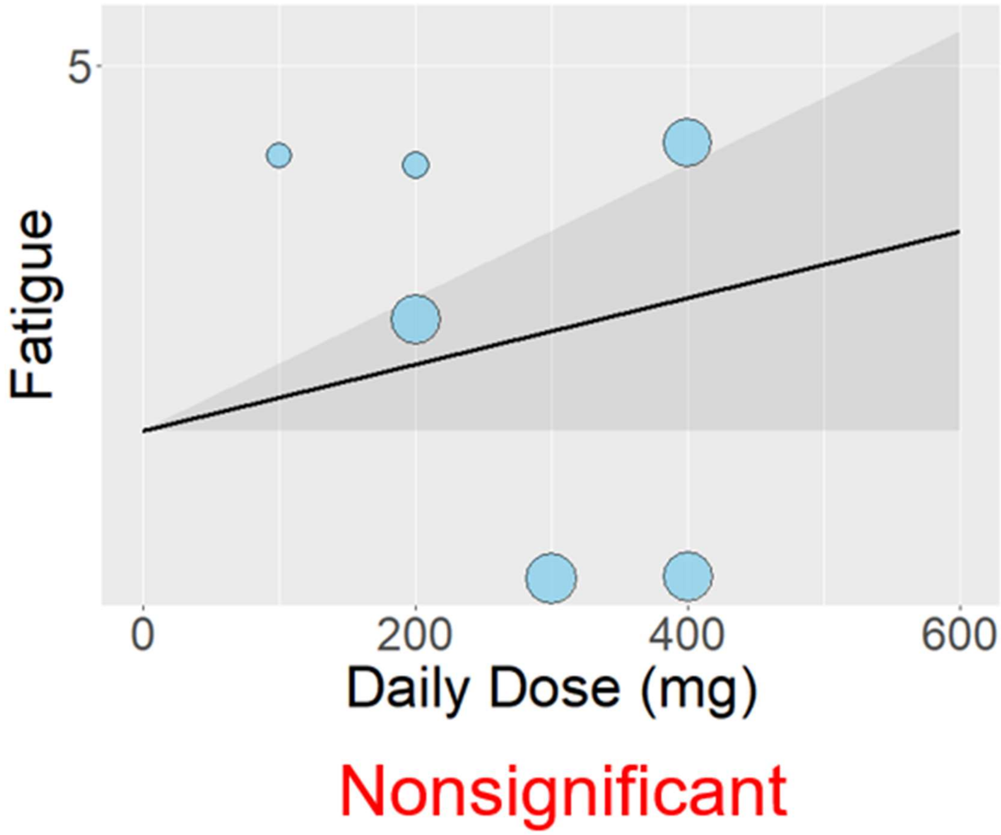

© 2024 Yu C et al. *JAMA Network Open.*

eFigure 14. Dose Response Meta-Analysis for Risk Ratio of Headache

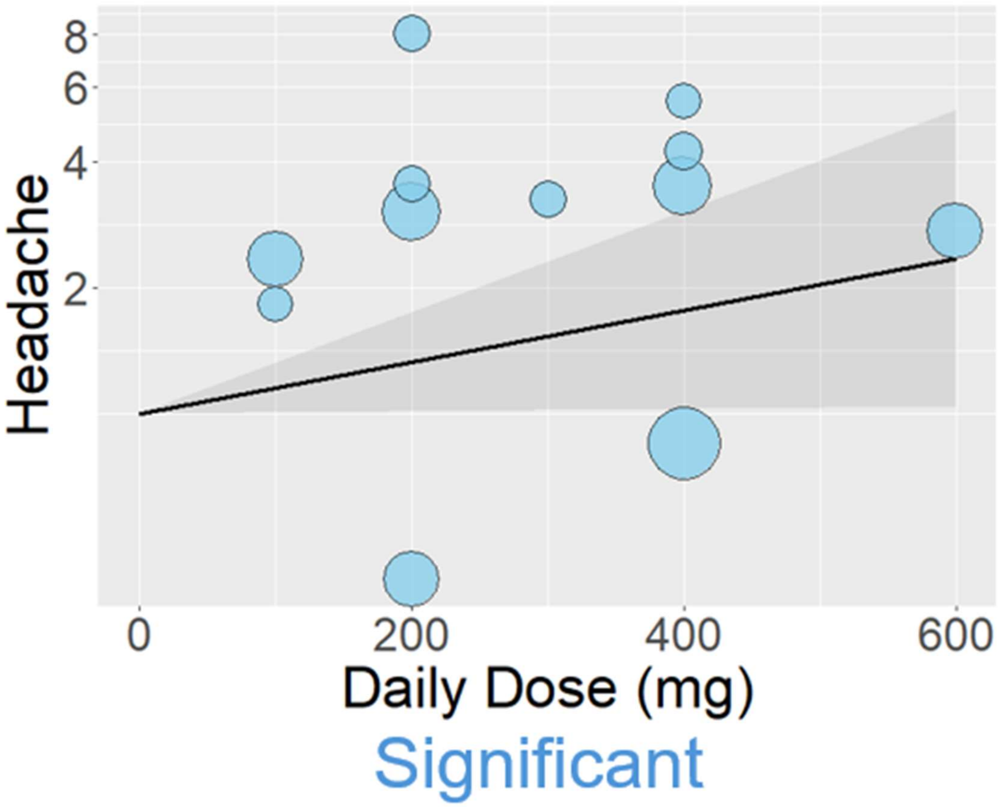

© 2024 Yu C et al. *JAMA Network Open.*

eFigure 15. Dose Response Meta-Analysis for Risk Ratio of Poor Appetite

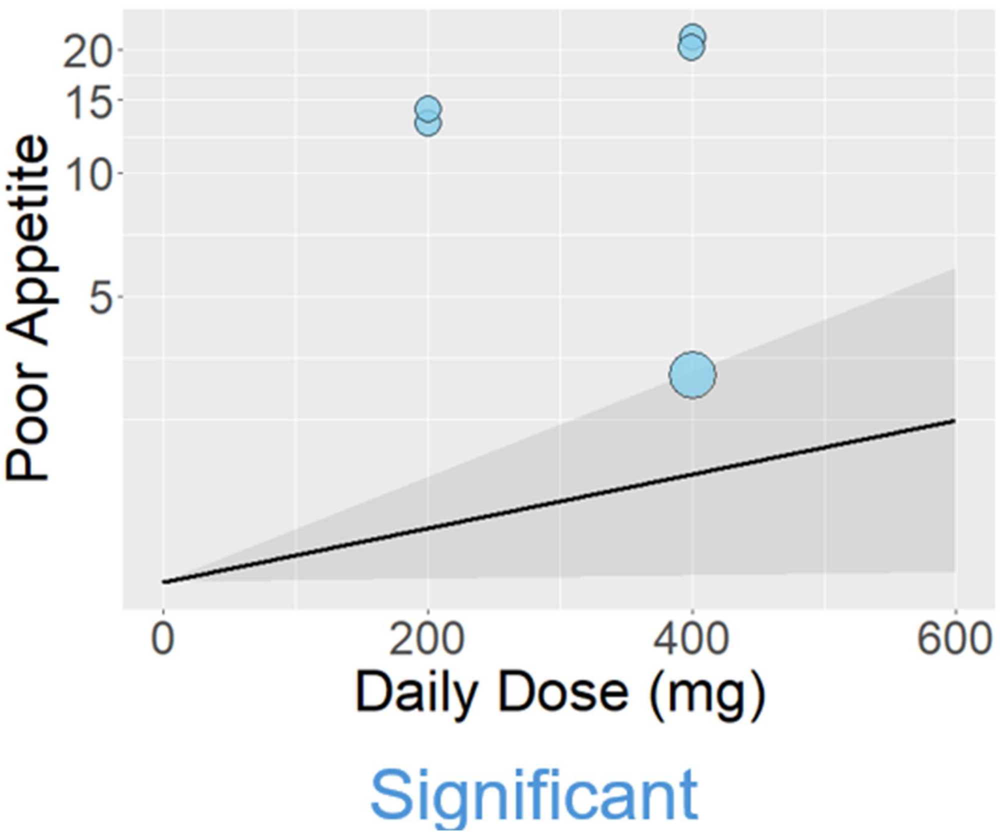

© 2024 Yu C et al. *JAMA Network Open.*

eFigure 16. Dose Response Meta-Analysis for Risk Ratio of Nausea

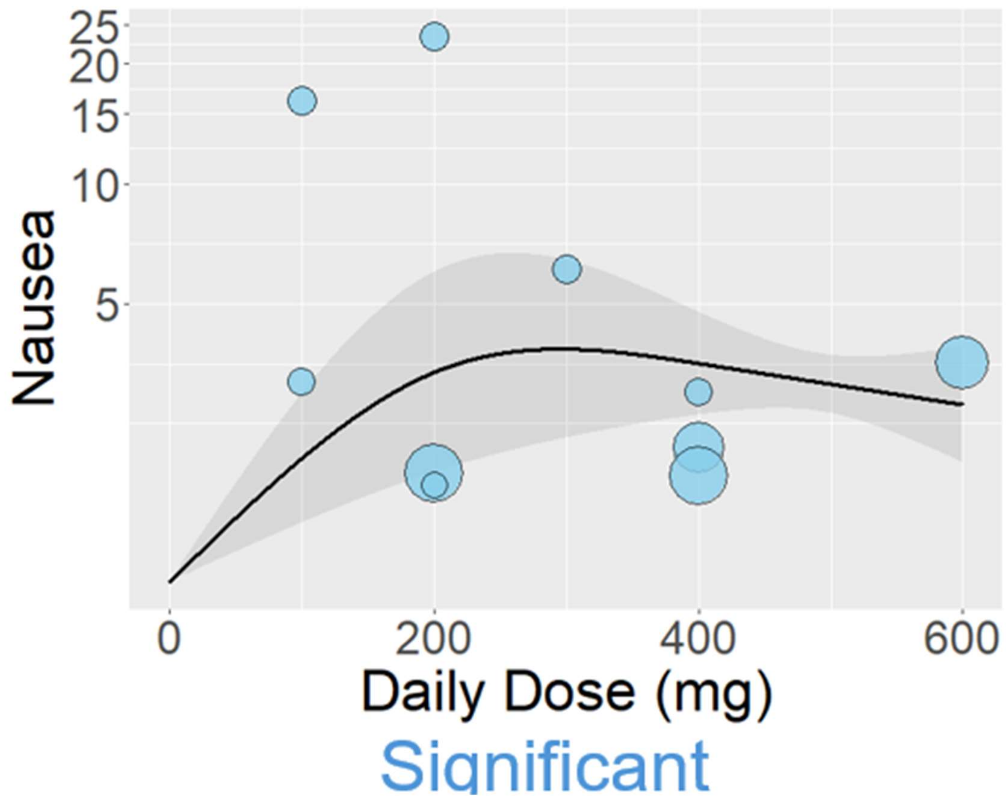

© 2024 Yu C et al. *JAMA Network Open.*

eFigure 17. Dose Response Meta-Analysis for Risk Ratio of Somnolence

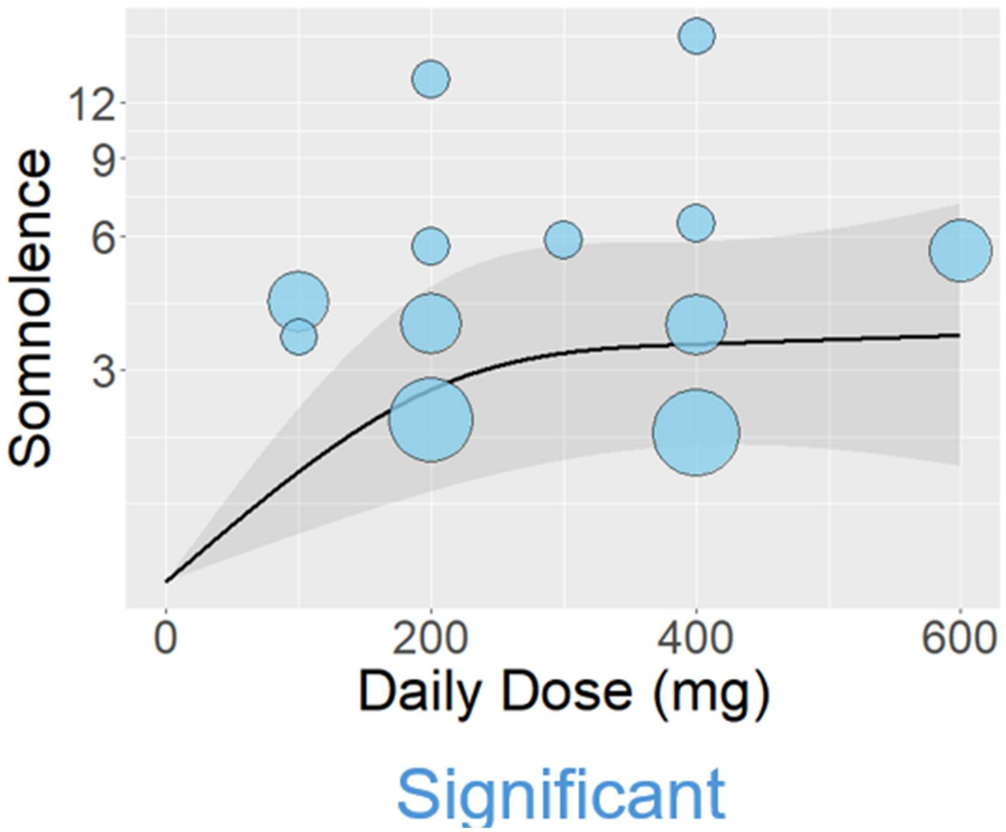

© 2024 Yu C et al. *JAMA Network Open.*
