# Supplementary material for: Response Trajectories and Temporal Trends of Viloxazine Treatment for Young People With ADHD: A Meta-Analysis
Source: JAMA Netw Open. 2024 Nov 19;7(11):e2445885. doi: 10.1001/jamanetworkopen.2024.45885 (PMC11577144; doi:10.1001/jamanetworkopen.2024.45885)
Supplement: Supplement 2. — Data Sharing Statement [file jamanetwopen-e2445885-s002.pdf]

Data Sharing Statement

Yu. Response Trajectories and Temporal Trends of Viloxazine Treatment for Young People With ADHD. *JAMA Netw Open*. Published November 19, 2024.
doi:10.1001/jamanetworkopen.2024.45885

Data
Data available: No

Additional Information
Explanation for why data not available: This is a meta-analytic study. The data were extract from other clinical trials
